# Decoding a Signature-Based Model of Transcription Cofactor Recruitment Dictated by Cardinal Cis-Regulatory Elements in Proximal Promoter Regions

**DOI:** 10.1371/journal.pgen.1003906

**Published:** 2013-11-07

**Authors:** Christopher Benner, Sergiy Konovalov, Carlos Mackintosh, Kasey R. Hutt, Rieka Stunnenberg, Ivan Garcia-Bassets

**Affiliations:** 1 The Salk Institute for Biological Studies, La Jolla, California, United States of America; 2 Department of Medicine, School of Medicine, University of California, San Diego, La Jolla, California, United States of America; Stanford University School of Medicine, United States of America

## Abstract

Genome-wide maps of DNase I hypersensitive sites (DHSs) reveal that most human promoters contain perpetually active cis-regulatory elements between −150 bp and +50 bp (−150/+50 bp) relative to the transcription start site (TSS). Transcription factors (TFs) recruit cofactors (chromatin remodelers, histone/protein-modifying enzymes, and scaffold proteins) to these elements in order to organize the local chromatin structure and coordinate the balance of post-translational modifications nearby, contributing to the overall regulation of transcription. However, the rules of TF-mediated cofactor recruitment to the −150/+50 bp promoter regions remain poorly understood. Here, we provide evidence for a general model in which a series of cis-regulatory elements (here termed ‘cardinal’ motifs) prefer acting individually, rather than in fixed combinations, within the −150/+50 bp regions to recruit TFs that dictate cofactor signatures distinctive of specific promoter subsets. Subsequently, human promoters can be subclassified based on the presence of cardinal elements and their associated cofactor signatures. In this study, furthermore, we have focused on promoters containing the nuclear respiratory factor 1 (NRF1) motif as the cardinal cis-regulatory element and have identified the pervasive association of NRF1 with the cofactor lysine-specific demethylase 1 (LSD1/KDM1A). This signature might be distinctive of promoters regulating nuclear-encoded mitochondrial and other particular genes in at least some cells. Together, we propose that decoding a signature-based, expanded model of control at proximal promoter regions should lead to a better understanding of coordinated regulation of gene transcription.

## Author Summary

Human cells exploit different mechanisms to coordinate the expression of both protein-coding and non-coding RNAs. Elucidating these mechanisms is essential to understanding normal physiology and disease. In our attempt to identify new regulatory layers acting particularly at proximal promoters, we have computationally analyzed the genomic sequences located from −150 bp to +50 bp relative to the transcriptional start site (TSS), which are often at the center of ‘open’ chromatin regions in human promoters. We have confirmed the presence of a series of cis-regulatory elements (here referred to as ‘cardinal’ motifs) that show a strong preference for these short regions. Interestingly, these elements tend to act independently rather than in fixed combinations. Therefore, we propose that they confer unique regulatory features to the human promoter subsets that contain each of these particular elements. In agreement with this model, we have identified a large repertoire of preferential partnerships between transcription factors recognizing cardinal motifs and their associated proteins (cofactors), thus decoding a signature-based model that distinguishes distinctive regulatory types of promoters based on cardinal motifs. These signatures may underlie a new layer of transcriptional regulation to orchestrate coordinated gene expression in human promoters.

## Introduction

DNase I hypersensitive sites (DHSs) mark ‘open’ chromatin regions in the human genome [1]. When profiled at genome-wide scale in many different tissues and cell types, DHS profiles reveal that most human promoters (at the transcriptional start site, TSS) remain in an ‘open’ chromatin state [2]–[4]. These ‘open’ chromatin areas center between −150 bp and +50 bp relative to the TSS (+1), although they could be larger depending on the mode of transcription initiation and identity of the specific promoter [2], [5]–[8]. They are also flanked by nucleosomes heavily modified with histone H3 lysine 4 dimethylation (H3K4me2) and trimethylation (H3K4me3), which also remain largely invariant across different cell and tissue types [2], [9]. Together, therefore, promoters (at the TSS) show a rather persistent chromatin organization that is likely associated with control of basal transcription [2], [9]–[13]. In fact, −40 bp to +40 bp regions (also known as ‘core’ promoters) generally act as entry sites for the pre-initiation complex (PIC) [5], [6], [14], and −150 bp to −40 bp regions (also known as ‘proximal’ promoters) contain abundant and conserved cis-acting regulatory elements that contribute to basal transcription [15]–[18].

The genome-wide profiling of transcription factors (TFs) and cofactors (i.e. TF-associated factors that do not bind to DNA and that often act as chromatin remodeling activities, histone/protein-modifying enzymes, or scaffold proteins) has recently provided valuable information that may change our understanding of how chromatin organization is established in promoters. Proximal promoters have been traditionally viewed as the main targets of TFs in the human genome; however, most TF binding profiles consistently reveal preferential binding to distal, rather than proximal, genomic sites (e.g. [19]–[23]). In an apparent paradox, many cofactors (such as histone/protein-modifying activities) often show preferential binding to promoter, rather than distal, genomic sites, which is more consistent with the traditional view that promoters are the major recruiters of transcriptional regulators in the genome [24]–[32]. In some cases, it has been proposed that histone/protein-modifying activities may directly recognize promoter-specific cis-regulatory elements, such as in the case of JARID2 and KDM2A, which are two lysine demethylase activities (KDMs) that remove methyl groups from lysine residues and directly recognize GC-rich sites [24], [33], [34]. GC-rich sites are common at proximal promoters [35], [36]. However, it is unclear how a highly abundant TF at promoters, such as Sp1, which binds with high affinity to GC-rich sites, functions to facilitate or compete with the binding of these cofactors at these regions. The same question stands for other abundant proximal promoter TFs (e.g. [12], [37]–[41]). It is also unclear the recruitment in most of other cases in which histone/protein-modifying activities do not recognize DNA. Together, preventing a clear picture of how TF binding patterns relate to those of histone/protein-modifying enzymes at promoter regions.

Here, we have analyzed 21,000 human promoters from −150 bp to +50 bp relative to the TSS to investigate the role of cis-regulatory elements and their cognate TFs in recruiting histone/protein-modifying activities, particularly KDMs, to these sites. Co-occurrence analysis of the most highly enriched of these elements (here termed ‘cardinal’ motifs) confirms that they tend to occupy these regions in patterns that are independent from one another, thus suggesting the existence of promoter subclasses based on the independent presence of these motifs. To validate this model, we profiled NRF1 and subunit B of NFY (NFYB), which constitutively recognize two of the most abundant cardinal elements, NRF1 and NFY/CCAAT. Our data confirmed that both ‘cardinal TFs’ (for recognizing cardinal motifs) occupy two largely independent promoter subsets. Furthermore, we screened for KDM activities that may selectively act via one TF but not the other, finding that LSD1 acts as a specific and pervasive cofactor of NRF1. We further explored the binding profiles of approximately 60 other cofactors reported in the literature, which resulted in the identification of other strong cardinal motif-cofactor signatures. Together, we propose that an important function of cardinal cis-regulatory elements at promoter DHSs is to dictate a selective regulatory code of histone/protein-modifying activities and other cofactors that distinguishes promoter subclasses. Intriguingly, each subclass shows qualitative and quantitative differences with regard to the type and number of cofactors recruited, thus suggesting that there is a complex regulatory layer depending on the presence of cardinal elements that might contribute to the chromatin organization and regulation of DHS promoters.

## Results

### Poor co-occurrence among motifs highly enriched at −150/+50 bp regions

**Figure 1:**
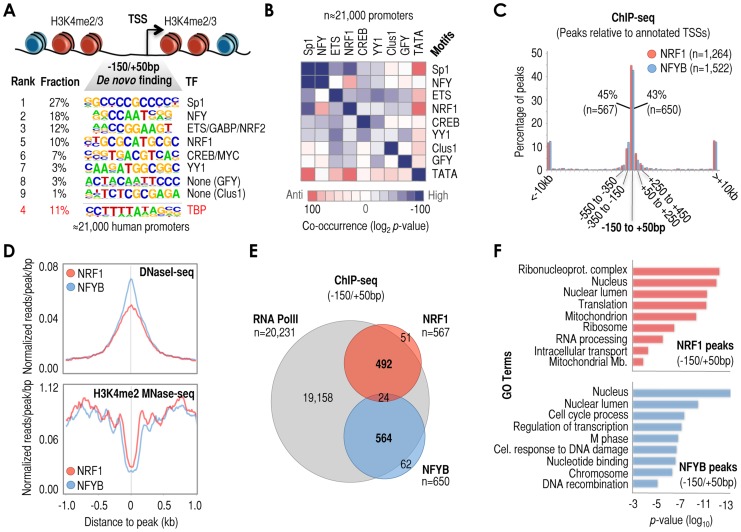
A series of cis-regulatory elements (here termed ‘cardinal’ motifs) are highly enriched at −150/+50 bp relative to TSS (+1) and may define different subsets of human promoters. (***A***) Most human promoters contain ‘open’ chromatin regions at −150/+50 bp relative to +1, or TSS. These regions are surrounded by heavily modified nucleosomes containing H3K4me2/3 (depicted in red in the vignette). We have identified the most enriched cis-regulatory elements in these particular regions by *de novo* motif discovery analysis of n = 21,000 human promoters. The panel shows rank of element enrichment, fraction of promoters containing these elements, consensus sequence, and cognate TF when known (e.g. NRF1 or NFY) or when proposed (e.g. Clus1). We refer to these elements as ‘cardinal’ motifs, and to the TFs that recognize them as ‘cardinal’ TFs. (***B***) Analysis of motif co-occurrences among cardinal motifs. Results are shown as a matrix of co-occurrences based on the analysis of n = 21,000 human promoters (−150/+50 bp). Co-occurrence log_2_ *p*-values are shown as a gradient of blue-to-red for positive-to-negative co-occurrences, and as white in the absence of significant co-occurrence. (***C***) Positional binding analysis of cardinal TFs NRF1 (red) and NFYB (blue) with respect to −150/+50 bp genomic regions in MCF7 cells, based on ChIP-seq data. The x-axis refers to genomic distances with respect to −150/+50 bp (center of the panel). Genomic windows span: 200 bp (between −150/+50 bp and ±2 kb), 1 kb (between ±2 kb and ±10 kb), and the rest of distances together (beyond ±10 kb). The y-axis refers to percentage of the total of NRF1 and NFYB peaks in each genomic range. The total number of peaks (n) and the specific number of peaks within −150/+50 bp regions (n) are also indicated in the panel. (***D***) Meta-analysis of sequencing read density based on DNaseI-seq (top) and H3K4me2 MNase-seq (bottom) around NRF1 (red) and NFYB (blue) ChIP-seq peaks (both at the center of the panel). (***E***) Venn diagram depicting the overlap of RNA PolII (grey circle), NRF1 (red circle), and NFYB (blue circle) ChIP-seq peaks in MCF7 cells. We considered as ‘overlap’ the coincidence of NRF1 and NFYB peaks in the same −150/+50 bp region. Also, we considered as ‘overlap’ the coincidence of RNA PolII peaks within ±1 kb of a TSS containing NRF1 or NFYB peaks at −150/+50 bp. *(* ***F*** *)* Functional (gene ontology, or GO) analysis of genes with NRF1 (top) or NFYB (bottom) ChIP-seq peaks in their −150/+50 bp regions. *P*-values (log scale) are shown in the *x*-axis. GO terms are indicated in the y-axis.

To guide the discovery of new regulatory mechanisms acting via core/proximal promoter regions, we analyzed −150/+50 bp regions (relative to the TSS) based on previous studies showing that these genomic coordinates overlap with the center of DNase I hypersensitivity in active human promoters [2], and accumulate promoter-specific motifs [37], [38], [42]–[45]. We extracted ‘all’ −150/+50 bp regions in the human genome (n = 21,000; independently of their chromatin state in a particular cell or condition) and performed *de novo* motif discovery analysis. This analysis resulted in the identification of nine highly enriched motifs, which we defined as ‘cardinal’ cis-regulatory elements. As expected, these nine elements included the TATA-box, as well as sequences recognized by well know ubiquitous TFs common in promoters: Sp1/GC-rich, NFY/CCAAT-box, ETS/GABP/NRF2, NRF1, CREB/CRE-MYC/E-box, and YY1 (**Figure 1A**). We also identified two cardinal motifs whose recognition by specific TFs has been poorly established: Clus1 [37], which may act as binding site for the zinc finger TF Kaiso/ZBTB33 [46]; and a sequence that we named GFY (for general factor Y), which may act as a binding site for the TFs Ronin/Hcf-1 and Zfp143/Rbp-J [46], [47] (**Figure 1A**). Co-occurrence analysis of these nine elements showed differential patterns of co-enrichment. For example, each of these sequences (with the only exception of YY1) showed a higher tendency to co-exist with copies of itself than with copies of the other eight cardinal elements within the same −150/+50 bp region (see the dark blue squares mostly in the diagonal in **Figure 1B**). Therefore, our analysis indicates that −150/+50 bp regions are more likely occupied by a single type of element rather than by fixed combinations of different cardinal elements. Only in the case of the NFY/CCAAT-box and the Sp1/GC-rich motifs did we observe high tendencies to co-occur (**Figure 1B**; summarized in **Figure S1A**). In addition, we performed co-occurrence analysis using experimentally defined promoter DHSs in breast cancer MCF7 cells and derived essentially the same conclusion, although positive co-occurrences between different cardinal motifs were even less significant (**Figure S1B**).

To test the predictive value of these analyses we focused on motifs other than TATA-box and Sp1/GC-rich because these two are well documented in the literature. Thus we performed chromatin immunoprecipitation followed by massively parallel sequencing (ChIP-seq) using antibodies that recognize nuclear respiratory factor 1 (NRF1, or alpha-PAL), which binds as a homodimer to the NRF1 site [48] and the nuclear transcription factor Y (NFY, or CBF), which binds as an obligatory heterotrimer of NFYA, NFYB, and NFYC to the NFY/CCAAT site [49]. Based on the computational analysis, we predicted that NRF1 and NFY would only occasionally coincide at −150/+50 bp regions (**Figures 1B** **and S1B**). ChIP-seq analysis in MCF7 cells revealed 1,264 and 1,522 high confidence NRF1 and NFYB peaks, respectively (**Supplementary Tables S1** and **S2**), and as expected, these peaks were found preferentially at promoter regions (**Figure S1C**), particularly within −150/+50 bp regions (43–45%, **Figure 1C**), also at the center of DHS and at the nucleosome-free or -depleted region (NFR/NDRs) (**Figure 1D**), and being surrounded by nucleosomes containing H3K4me2 and H3K4me3 (**Figures 1D** **and S1D**). We also performed analyses of ChIP-seq datasets available in the literature (although in some cases in other cell lines) and established a similar relationship between the set of predicted motifs, the actual TF peaks, experimentally defined DHSs, and the profiles of histone marks H3K4me2 and H3K4me3 (**Figure S1E**). Importantly, -returning to the case of NRF1 and NFYB- both TFs rarely co-localized at −150/+50 bp (∼4%, **Figure 1E**), consistent with our prediction. This low rate of co-binding of NRF1 and NFYB did not substantially increase upon examining wider promoter regions (7–8% between −800 bp and +200 bp, **Figure S2A**), thus confirming their apparent binding antagonism in promoters across the human genome.

Since NRF1 and NFYB occupy only 25% and 18% of their respective predicted sites at −150/+50 bp regions (based on the comparison of ChIP-seq data and computational prediction of NRF1 and NFY sites), we could not exclude the possibility that their poor co-localization may be a result of technical limitations associated with the ChIP-seq assay. To assess this possibility, we alternatively assessed genomic binding of NRF1 and NFYB using the highly sensitive ChIP-DSL assay [50]. This assay, in contrast to ChIP-seq, is a targeted approach that lacks the direct amplification of ChIP'ed DNA (see Methods for more details). Using the Hu20K array (which allows for the targeted testing of ∼20,000 human promoters between −800 bp and +200 bp relative to the TSS), we re-identified 63–73% of the NRF1 and 72–81% of the NFYB ChIP-seq-positive promoters (depending on how stringently we defined a ChIP-DSL-positive hit: p<0.0001-p<0.01; **Figure S2B**). Using the most stringent analysis (p<0.0001), the ChIP-DSL assay identified large subsets of NRF1 and NFYB positive promoters that were not identified by ChIP-seq (1,320 and 1,525, respectively; **Figure S2B**), which were also highly enriched in NRF1 or NFY/CCAAT motifs. However, consistent with our ChIP-seq analyses, they showed relatively poor NRF1 and NFYB co-localization (**Figure S2C**). The only exception to this observation was a small subset of NRF1 and NFYB co-occupied promoters (n = 332, **Figure S2C**), in which NRF1 and NFY/CCAAT motifs also co-occurred with abnormal high significance (**Figure S2D**). Overall, therefore, our ChIP-seq and ChIP-DSL results with NRF1 and NFYB confirm the predictive value of our computational analysis, which suggests that cardinal motifs (and their cognate TFs) tend to be present independently rather than in fixed pairs within these regions. In fact, combined analysis of the NRF1 and NFYB ChIP-DSL datasets showed that almost 35% of all human promoters tested on the Hu20K array contain one or the other TF, although they still poorly coincide. We also performed gene ontology (GO) analysis of genes associated with −150/+50 bp NRF1 and NFYB ChIP-seq peaks to associate them with biological functions. As expected, based on the known functions of NRF1, genes associated with this TF were linked to RNA processing and metabolism, translation, mitochondria, and intracellular transport of proteins (**Figure 1F**, top panel), whereas those associated with NFYB were linked to cell cycle, regulation of transcription, and response to DNA damage, among others (**Figure 1F**, bottom panel). Similar results were obtained using the ChIP-DSL data (**Figure S2E**). Therefore, the tendency to occupy different promoters may also be associated with their specific biological functions.

### Cardinal cis-regulatory elements dictate regulatory signatures of histone-modifying enzymes

**Figure 2:**
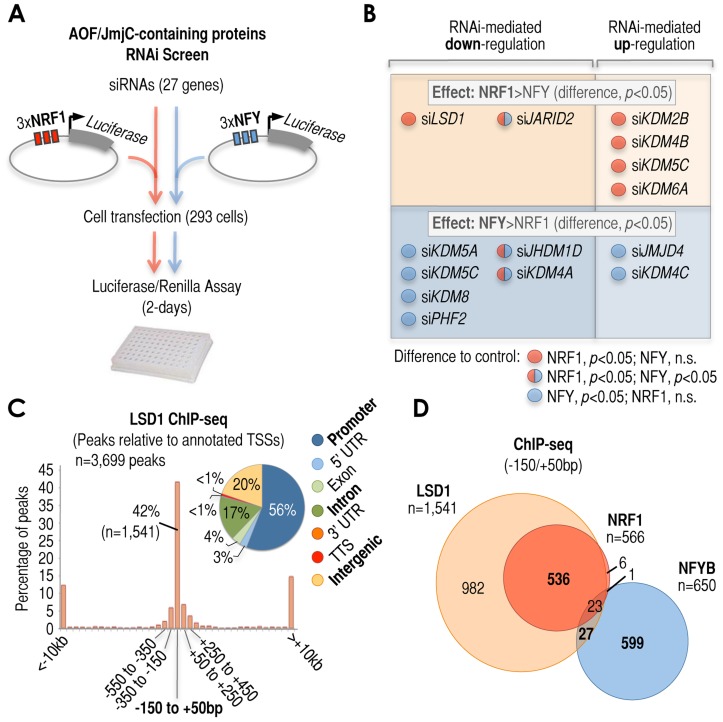
Cardinal motifs dictate patterns of transcriptional regulatory activities. *(* ***A*** *)* Schematic overview of the siRNA-based screen to identify lysine demethylase (KDM) activities that may act selectively via NRF1 or NFYB motifs. KDMs belong to two gene families: the family of amine oxidase flavin (AOF)-containing domain proteins, and the family of Jumonji C (JmjC)-containing proteins. HEK293T cells were transfected with a vector expressing the luciferase gene under control of three multimerized copies of NRF1 (3×NRF1, left side of the scheme) or three multimerized copies of NFY (3×NFY, right side of the scheme). Luciferase levels were tested after independent treatment with n = 27 different KDM siRNAs, plus controls. *(* ***B*** *)* Summary of siRNA-mediated effects that showed selectivity for 3×NRF1 or 3×NFY motifs in our screen. A total of n = 14 out of n = 27 siRNA treatments induced selective effects: n = 6 were selective of the presence of 3×NRF1 (top), and n = 8 were selective of the presence of 3×NFY (bottom). Of these n = 14 siRNA treatments, furthermore, n = 8 induced down-regulation of the reported gene (left), and n = 6 induced its up-regulation (right). Selectivity for 3×NRF1 or 3×NFY motifs was established by the direct comparison of siRNA-mediated effects induced by the same siRNA treatment on 3×NRF1- or 3×NFY-luciferase ((*p*<0.05; last row in **Figure S3A**). In n = 11 out of the n = 14 treatments (red circles if selective of 3×NRF1, or blue circles if selective of 3×NFY), the difference between the specific KDM and control (scrambled) siRNA-mediated effect was also statistically significant (*p*<0.05; first and second rows in **Figure S3A**). In the other n = 3 out of the n = 14 selective siRNA treatments (red-and-blue circles), the difference with respect to control siRNA was statistically significant for both 3×NRF1- and 3×NFY-regulated units, although statistically significant also when compared between them (*p*<0.05; last row in **Figure S3A**). *(* ***C*** *)* Positional binding analysis of LSD1 in MCF7 cells (as in **Figure 1C**), and genomic localization of LSD1 ChIP-seq peaks with respect to the genome annotation (pie chart). The numbers included in the pie chart refer to the fraction of LSD1 peaks associated with each annotated region. *(* ***D*** *)* Venn diagram depicting the overlap of LSD1 (orange), NRF1 (red), and NFYB (blue) ChIP-seq peaks at −150/+50 bp regions in MCF7 cells. We considered as ‘overlap’ the coincidence of NRF1, NFYB, and/or LSD1 peaks in the same −150/+50 bp region.

If many −150/+50 bp regions could be distinguished by a single cardinal cis-regulatory element and its cognate TF, then we hypothesized that this element could also lead to selective (distinguishable) recruitment of cofactors via TFs. To test this hypothesis, we focused on KDMs because these histone-modifying enzymes have been repeatedly shown to bind preferentially to promoters in genome-wide tests [24], [26]–[31], although their rules of TF-mediated recruitment to promoters are poorly understood, especially on a genome-wide scale [51], [52]. In order to elucidate the role of KDMs on NRF1- and NFYB-mediated transcription, we tested the effects of short interfering RNA (siRNA)-mediated depletion of KDMs on luciferase transcription under the control of three canonical NRF1 sites (3×NRF1-*Luc*) or three canonical NFY sites (3×NFY-*Luc*) in HEK293T cells (**Figure 2A**). Although these sites are not in the context of endogenous promoters (thus results should be taken with caution), this minimalist strategy ensures that the only difference between these two promoters is the cardinal motif. We tested 27 siRNAs that corresponded to the 27 KDMs that are expressed in these cells (based on gene expression profiles), out of around 30 encoded in the human genome. KDM specificity for NRF1 or NFY sites was established by comparing the relative effects of the same KDM siRNA treatment on NRF1- and NFY-regulated luciferase transcription with respect to control siRNA (**Figure S3A**). By performing these comparisons, we identified 13 siRNA treatments that had selective influences (*p*-value<0.05) on luciferase transcription depending on whether the motifs were NRF1 or NFY (summarized in **Figure 2B**). Six of these siRNAs specifically altered 3×NRF1-dependent transcription (**Figure 2B**, top), while 8 siRNAs specifically affected 3×NFY-dependent transcription (**Figure 2B**, bottom). One siRNA treatment (*KDM5C* siRNA) had significant effects on both 3×NRF1- and 3×NFY-dependent transcriptional units when compared to siRNA control, but with opposite effect on each reporter. Three other siRNAs (red/blue circles in **Figure 2B**) also induced changes in the expressions of both reporters when compared to siRNA control, however, these effects were in the same direction and clearly more significant for one site over the other (*p*-value<0.05 between them). Overall, 8 siRNA treatments induced down-regulation of gene expression (**Figure 2B**, left), while 6 induced up-regulation compared to control siRNA (**Figure 2B**, right), which may interestingly suggest that these cardinal motifs impose a balance of positive and negative activities on the same promoter, rather than an exclusive effect of a single activity. The results of this analysis also suggest that 3× copies of the same cardinal motif at −150/+50 bp regions are sufficient to dictate a rich and selective regulatory pattern of KDMs.

Because the results just described could be either the result of direct or indirect effects, we capitalized on our specific result revealing that *LSD1* siRNA alters NRF1- but not NFY-motif mediated transcription (**Figure 2B**, top and left) by using the case of LSD1 to study cardinal motif-induced KDM regulatory patterns in more detail. For our study, we performed ChIP-seq analysis of lysine specific demethylase 1 (LSD1) in MCF7 cells, identifying 3,690 high confidence peaks that showed high preference for annotated promoter regions (**Figure 2C**, pie chart). A full list of genomic locations can be found in **Supplementary Table S3**. LSD1 shows its strongest binding preference for the −150/+50 bp region (42%, **Figure 2C**), at the center of the promoter DHS and NFR/NDR (**Figure S3B**). Surprisingly, this reveals that LSD1 is a genuine promoter DHS-specific factor, similar to NRF1 and NFYB (**Figure 1C**). When comparing the binding patterns of LSD1, NRF1, and NFYB, we observed that virtually all NRF1-positive regions were occupied by LSD1 (99%), with only a few NFYB-positive regions being occupied by LSD1 if NRF1 was not nearby (4%, **Figure 2D**). We also observed similar results for LSD1 promoter occupation using the highly sensitive ChIP-DSL assay (**Figure S3C**), as well as a high correlation of LSD1 binding with NRF1 on a genome-wide scale (**Figure S3D**). In the context of a third ‘cardinal’ TF, Sp1, we also observed a strong associative preference for LSD1 contingent on co-localization with NRF1 in a limited analysis of ∼2,000 human promoters (**Figure S3E**). Not surprisingly, *de novo* motif discovery analysis of LSD1 ChIP-seq peaks revealed overwhelming enrichment of NRF1 sites (**Figure S4A**). We also observed enrichment of the estrogen responsive element (ERE) in agreement with our own previous studies showing that estrogen receptor alpha (ERα) recruits LSD1 to ERα-regulated regions via EREs [53]. However, the co-localization of ERα and LSD1 was mostly found at distal (non-promoter or H3K4me3-negative) sites (**Figure S4B** and **S4C**). In contrast, the co-association between NRF1 and LSD1 was characteristic of promoter (or H3K4me3-positive) regions (**Figure S4C**).

Next, we examined whether strong NRF1 and LSD1 co-association is a cell type-specific feature. We performed LSD1 ChIP-DSL analyses in human mammary epithelial (HMEC), prostate cancer (LNCaP), osteosarcoma (U2OS), and neuroblastoma (SH-SY5Y) cells. Our results showed significant NRF1 motif enrichment in LSD1 peaks in all four examined cell lines, although the levels of enrichment were slightly different among them (**Figure S5A**). U2OS cells showed almost identical motif enrichment to that observed in MCF7 cells (**Figure S5A**), and more than 80% of the LSD1-positive promoters in these cells were also LSD1-positive in MCF7 cells. Based on this finding, we included U2OS cells in some of the experiments reported later in this study. To confirm the high binding coincidence of LSD1 in U2OS and MCF7 cells, we performed standard ChIP analysis on random targets (**Figure S5B**), and on a few classic NRF1-regulated promoters (**Figure S5C**), and observed almost identical LSD1 binding patterns in both cell lines. The LSD1 binding program has also been recently reported in mouse embryonic stem cells (mESCs) [54], and although no particular connection between LSD1 and NRF1 was highlighted in this study, we analyzed the available data and observed that the NRF1 site is also significantly enriched between -150 bp and +50 bp relative to the TSS in these cells (p = 1e-42). In fact, the LSD1 binding map in human MCF7 and mouse ESCs differs at many sites (which is expected, especially at distal regions, since these two lines derive from different organisms and are completely different in many aspects [55]), but they show remarkably similar binding profiles at many TSSs, including at those of classic NRF1 target promoters (**Figures S5D** and **S6**). Taken together, our results show that the strong co-association of NRF1 motifs, NRF1 TF, and the LSD1 cofactor at −150/+50 bp regions can be observed in different cell lines and organisms, which supports a model in which cis-regulatory elements in these regions dictate strong and common cofactor signatures.

### Cardinal TFs recruit ‘signature’ cofactors

**Figure 3:**
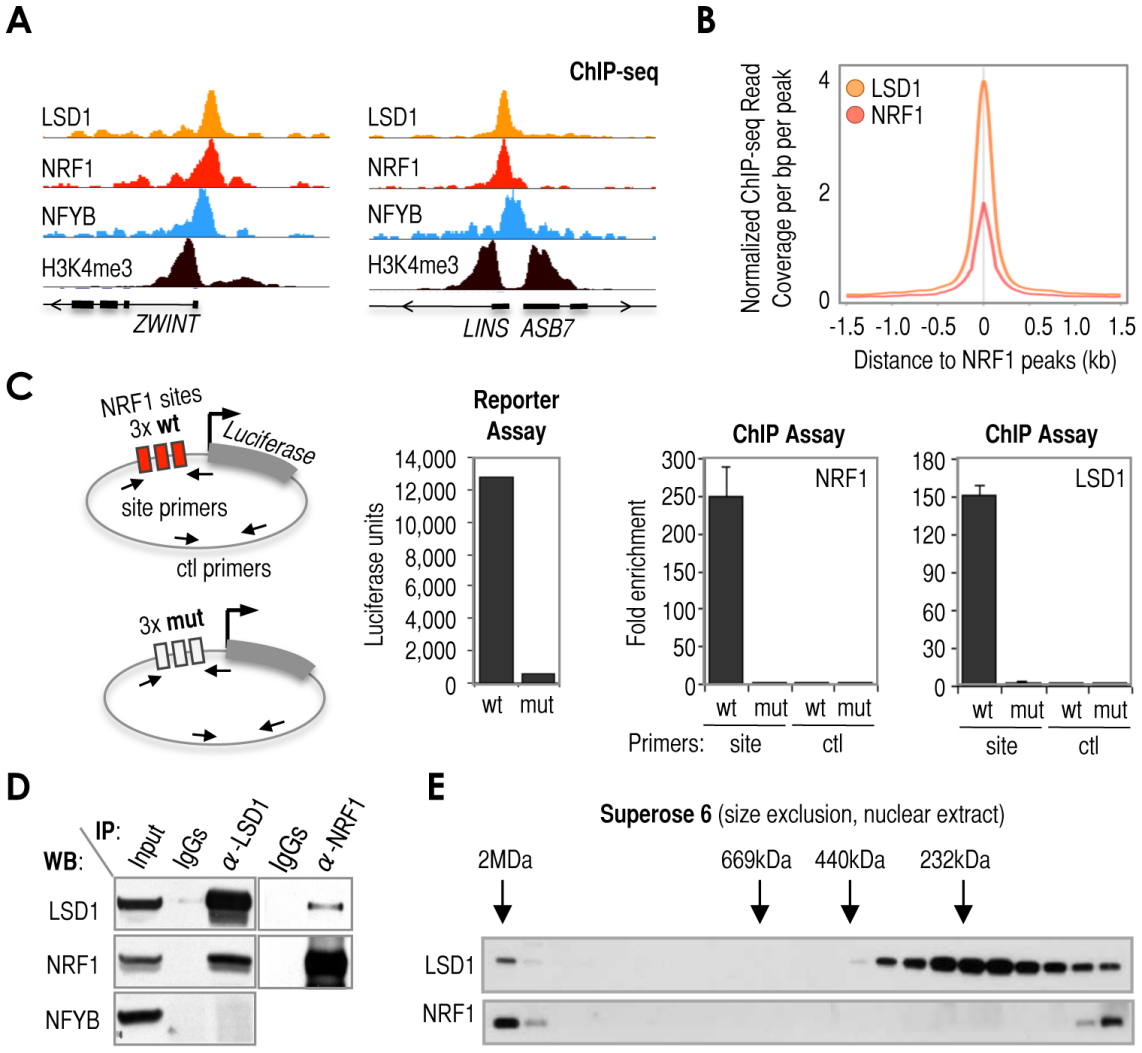
Cardinal motif NRF1 dictates the recruitment of KDM LSD1 via TF NRF1. *(* ***A*** *)* Representative examples of ChIP-seq tracks showing precise co-alignment of NRF1 and LSD1. These particular loci (*ZWINT* and *LINS-ASB7*) were selected as representative despite being rare examples in which NRF1 binds nearby NFYB, but they should help to emphasize the good co-alignment between LSD1 and NRF1 using NFYB as reference. The track of ChIP-seq data for H3K4me3 was also included as reference. Annotation of Ref-seq genes is included. *(* ***B*** *)* Meta-analysis of sequencing read density of LSD1 ChIP-seq (orange) and NRF1 ChIP-seq (red) signals around NRF1 peaks (center of the panel). *(* ***C*** *)* Left: Scheme of two constructs engineered to contain 3× wild-type NRF1 sequences (3xwtNRF1, as in **Figure 2A**) or 3× mutated NRF1 sequences (3xmutNRF1) upstream the luciferase reporter gene. Panels: Luciferase assay with 3xwtNRF1- or 3xmutNRF1-transected U2OS cells (left panel) and ChIP analyses of NRF1 and LSD1 at 3xwtNRF1 and 3xmutNRF1 sites (middle and right panels, respectively). For ChIP analyses, two regions were amplified (labeled in the scheme, left): one region covering 3xwtNRF1 or 3xmutNRF1 sites (depending on the construct), amplified with primers named as ‘site’; and the other region covering a distal, control area (the same in both constructs), amplified with primers named as ‘ctl’. *(* ***D*** *)* Co-immunoprecipitation (IP) assay with the set of antibodies indicated on top of the panel, and detection with the set of antibodies indicated in the left of the panel. *(* ***E*** *)* Size exclusion chromatography (Superose 6) of nuclear extracts obtained from MCF7 cells. Analysis of elution fractions by Western blot using anti-LSD1 (top) and anti-NRF1 (bottom) antibodies. On top of the Western blot, elution of known molecular size markers is indicated (arrows). Voided volume was determined with Blue Dextran 2000 (>2MDa).

Careful examination of LSD1 and NRF1 ChIP-seq peaks revealed their very close alignment at specific loci (**Figures 3A** **and S7**) and on a genome-wide scale (**Figure 3B**). To test whether NRF1 (indirectly, the NRF1 motif) could be responsible for recruiting LSD1 to a promoter region, we took advantage of the same luciferase expression system that we previously employed to test the involvement of KDMs in cardinal motif dependent transcription (**Figure 2A**). For these experiments, we engineered the construct containing the luciferase gene under control of 3×NRF1 sites (3×NRF1) to contain a sequence variant with point mutations expected to disrupt binding of NRF1 (scheme in **Figure 3C**, and Methods). Using the wild-type and mutated reporter constructs, we observed that both the levels of luciferase expression (measured by the luciferase assay) and NRF1/LSD1 binding (measured by standard ChIP) were completely dependent on the presence of wild-type NRF1 motifs, suggesting that LSD1 acts via NRF1 sites as a consequence of direct recruitment by NRF1 (**Figure 3C**). Also in support of this model, endogenous LSD1 co-immunoprecipitated with endogenous NRF1 and vice versa, whereas LSD1 did not co-immunoprecipitate with endogenous NFYB in the same cell extracts (**Figure 3D**). Size exclusion chromatography of nuclear extracts also suggested that NRF1 and LSD1 interact (directly or indirectly), since a pool of NRF1 co-fractionates with a pool of LSD1 as part of what could be a ‘super’-multiprotein complex of a molecular size larger than 2MDa (**Figure 3E**). We also observed an additional pool of NRF1 and LSD1 that co-fractionated in very slow elution fractions (**Figure 3E**), but these fractions likely corresponded to elution as individual molecules, rather than as physically associated partners. Taken together, these results suggest that NRF1, via NRF1 sites, could mediate the recruitment of LSD1 to promoter regions, which is consistent with their strong co-association on a genome-wide scale and in different cell types.

### Functional association of NRF1 and LSD1

**Figure 4:**
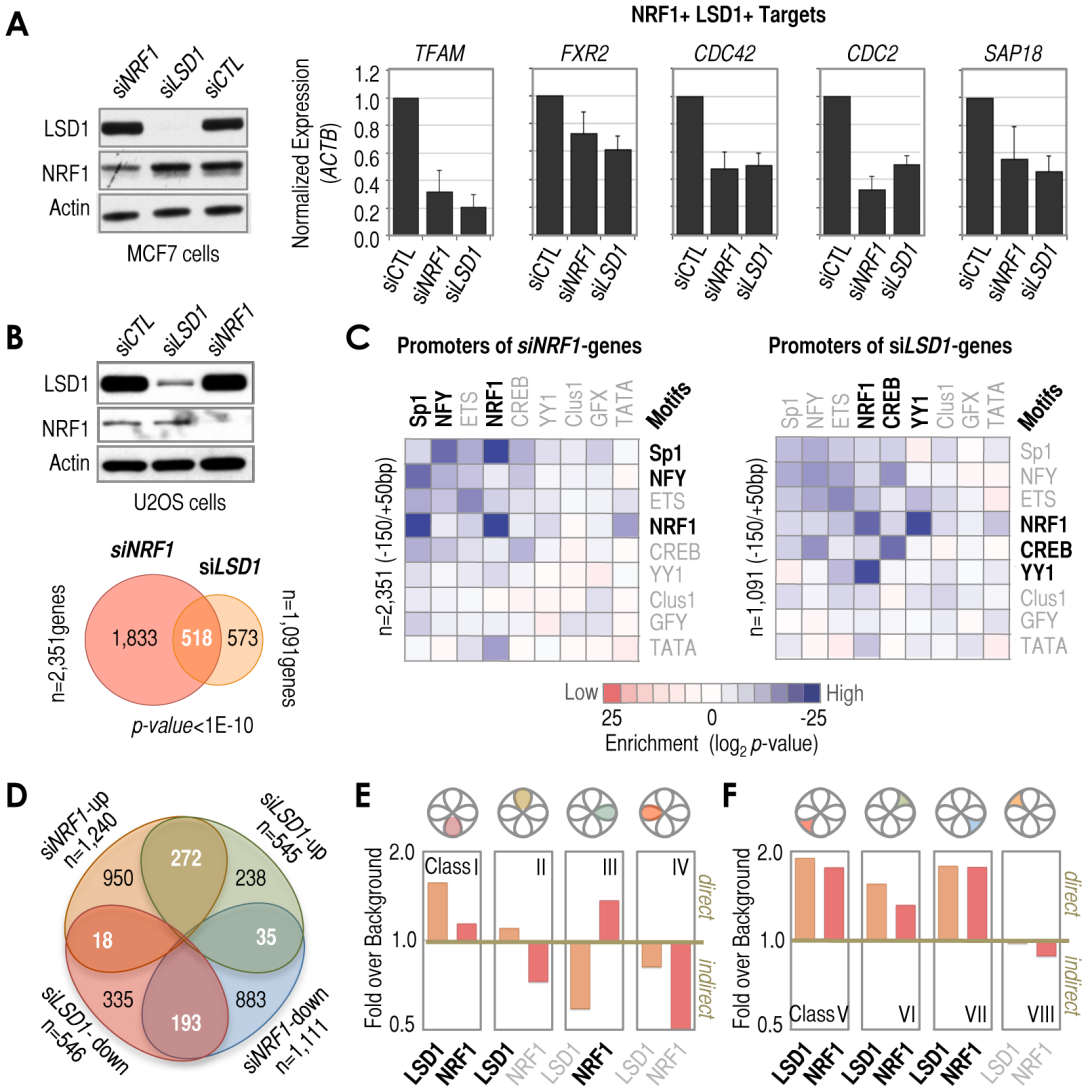
Diversity of functional outcomes associated with NRF1/LSD1 recruitment at −150/+50 bp regions. *(* ***A*** *)* Left: Western blot analysis of NRF1 and LSD1 in whole cell extracts obtained from MCF7 cells in which NRF1 or LSD1 (indicated on top) were depleted by siRNA. Actin is shown as loading control. Scrambled (CTL) siRNA is included as control of transfection. Panels: RT-qPCR analysis of genes that have been identified in this study as having promoters co-occupied by NRF1 and LSD1 (NRF1^+^ and LSD1^+^ targets). Gene names are indicated on top of each panel. Treatments are indicated at the bottom. Scrambled (CTL) siRNA was used as control. The y-axis refers to normalized expression to levels of *ACTB* mRNA. *(* ***B*** *)* Top: Western blot analysis as shown in ***A*** (left panel) but in U2OS cells. Bottom: Venn diagram depicting the overlap of genes affected by *NRF1* (light red circle) and *LSD1* (orange circle) siRNA treatments with respect to control (CTL) siRNA in U2OS cells, based on microarray. The number of total and category-wise genes affected by the treatments are indicated, as well as the statistical significance of the overlap. *(* ***C*** *)* Matrix of motif enrichment of cardinal motifs in −150/+50 bp regions of genes identified by microarray as affected by *NRF1* (left) or *LSD1* (right) knockdown in U2OS cells. Enrichment levels were determined with respect to background frequencies of the same motifs in −150/+50 bp regions. Motif enrichments higher than background are shown as a gradient of blue, while motif enrichments lower than background are shown as a gradient of red. No motif enrichment is shown as white. The number of promoters analyzed is also indicated. *(* ***D*** *)* Venn diagram depicting the overlap of microarray-identified genes classified based on their type of response to *NRF1* or *LSD1* siRNA treatments. The numbers of genes in the overlaps are indicated, as well as the numbers of genes that did not overlap and the total numbers. *(* ***E*** *)* Combined analysis of microarray and ChIP-based data. We combined microarray results identifying *NRF1* and/or *LSD1* siRNA-mediated effects and ChIP-DSL data identifying *NRF1* and/or *LSD1* occupied promoters. Gene classes based on (*D*). The y-axis refers to the relative enrichment over background in the number of genes that were affected by both *NRF1* and *LSD1* siRNA treatments and that had promoters occupied by LSD1 (orange) and/or NRF1 (red; see text for more details). A ratio of ‘fold over background’ higher or lower than 1 (>1 or <1, respectively) distinguishes when a gene class contains a higher or lower frequency of either LSD1- or NRF1-occupied promoters over the frequency observed in genes not affected by *LSD1* or *NRF1* siRNA treatments (which we defined as ‘background’). *(* ***F*** *)* As in ***E***, but for genes that were affected only by either *NRF1* or *LSD1* siRNA treatments.

The almost pervasive association of NRF1 with LSD1 at −150/+50 bp regions does not directly imply a functional relationship between them or, if such relationship exists, that it is functionally universal at every single promoter. In fact, any functional relationship between these two factors may be complex because LSD1 may act as either a coactivator or corepressor of transcription, depending on the context of its binding [56]–[60]. Our data obtained in the context of the 3×NRF1 sites suggested that LSD1 can act as coactivator of NRF1, at least under this ‘artificial’ condition (**Figure 2B**). To test this hypothesis on endogenous NRF1 sites, we tested two classic NRF1 targets (*TFAM* and *FXR2*) and three new NRF1/LSD1 targets uncovered in this study (*CDC42*, *CDC2*, and *SAP18*). For these five genes, both *LSD1* and *NRF1* knockdown resulted in decreased expression when compared to control siRNA, suggesting that LSD1 is in fact a coactivator of NRF1-mediated transcription (**Figure 4A**). To test this possibility on a genome-wide scale, we performed whole-genome expression profiling analysis following *NRF1*, *LSD1*, or control siRNA treatment. We identified 2,351 genes as significantly altered by *NRF1* knockdown, and 1,091 genes as significantly altered by *LSD1* knockdown, both compared to control siRNA. Of these genes, a very significant number of them were altered by both *LSD1* and *NRF1* siRNA treatments (n = 518, *p*-value<1.0E-10), or 22% and 47% of all *NRF1* and *LSD1* siRNA-affected genes, respectively (**Figure 4B**). About 90% of these 518 genes were affected in the same direction by both treatments (either up or down-regulated). Additionally, motif analysis of the −150/+50 bp regions associated with the *NRF1* and *LSD1* siRNA-altered genes showed high enrichment of NRF1 motifs, thus supporting the idea that many LSD1-functionally regulated genes (and obviously many NRF1-regulated genes) are *bona fide* NRF1 motif-containing promoters (**Figure 4C**). We were initially surprised that NFY/CCAAT motifs were also significantly enriched in the *NRF1* siRNA-altered genes (**Figure 4C**, left panel), however, we suspect that this enrichment may derive from the unexpected *NRF1* siRNA-mediated up-regulation of *NFYA* and *NFYB* genes (as determined by microarray), which are two components of the trimer that constitutes NFY, thus potentially affecting NFY/CCAAT motif-containing promoters indirectly.

Our genome-wide analysis supports the idea that NRF1 and LSD1 co-regulate gene transcription, but to establish the homogeneity or heterogeneity of this functional partnership we classified the full set of genes altered by both siRNA treatments into four classes: *Class I* included genes down-regulated by both siRNA treatments (n = 193); *Class II* included genes up-regulated by both siRNAs (n = 272); *Class III* included genes down-regulated by *NRF1* siRNA, but up-regulated by *LSD1* siRNA treatment (n = 35); and *Class IV* included genes up-regulated by *NRF1* siRNA, but down-regulated by *LSD1* siRNA (n = 18; **Figure 4D**). We also organized those genes only affected by one siRNA treatment into four classes (*Classes V–VIII*; **Figure 4D**). Next, to establish which transcriptional output is more likely to be associated with direct versus indirect NRF1/LSD1-dependent effects, we calculated the enrichment of NRF1- or LSD1-occupied promoters for genes in each class (based on ChIP-DSL data) and compared this value to the enrichment of NRF1- or LSD1-occupied promoters for genes not affected by *NRF1*/*LSD1* siRNA (which we defined as ‘background’). A ratio greater than one (>1) with respect to background might be associated with a higher frequency of direct effects mediated by the siRNA treatment, while a ratio lower than one (<1) might be associated with a higher frequency of indirect effects (since the rate of promoter binding in this case is lower than that observed in background, i.e. in siRNA-unaffected genes). Following our analysis, we observed that genes down-regulated by both *NRF1* and *LSD1* siRNAs tend to show a higher frequency of NRF1 and LSD1 binding at their promoters than background (**Figure 4E**, *Class I*). In contrast, genes up-regulated by both siRNA treatments tend to show a higher frequency of LSD1, but not NRF1, binding at their promoters (**Figure 4E**, *Class II*). The ratio obtained for genes down-regulated by *NRF1* siRNA and up-regulated by *LSD1* siRNA suggests a general enrichment in direct NRF1, but indirect LSD1 effects (**Figure 4E**, *Class III*), while the ratio obtained for genes up-regulated by *NRF1* siRNA and down-regulated by *LSD1* siRNA suggests that both NRF1 and LSD1 affect these genes indirectly (**Figure 4E**, *Class IV*). These results are consistent with the current view that NRF1 binds to promoters to activate gene transcription (*Classes I*, *III*, and *VII*), and that LSD1 either acts as a co-activator (*Classes I* and *V*) or a co-repressor of transcription (*Classes II* and *VI*). The only classes in which both NRF1 and LSD1 show a tendency to co-bind are *I*, *V*, *VI*, and *VII*, thus suggesting that in the context of the whole human genome, LSD1 may act as a NRF1 coactivator in some cases (*Class I*), or remain inactive in others (*Class VII*), at least under the experimental conditions that we tested. *Class VI* represents an interesting case in which LSD1 may inhibit the NRF1 activity, thus *LSD1* knockdown impairs the negative effect on NRF1-mediated activation, but *NRF1* knockdown *per se* has no effect on gene expression under already the condition of LSD1-mediated NRF1 inhibition. More difficult to explain are the *Class V* promoters. In this case, it is possible that other TFs (besides NRF1) may recruit LSD1 to these promoters, even if NRF1 is present, thus resulting in LSD1-dependent genes that are associated with, yet functionally independent of NRF1. Overall, this functional analysis suggests that even if a motif/TF/cofactor signature is pervasive in promoters across the genome, the functional relevance could be rather complex, thus emphasizing that these signatures should not be interpreted as representative of universal functional outcomes.

### A collection of regulatory signatures associated with cardinal motifs

**Figure 5:**
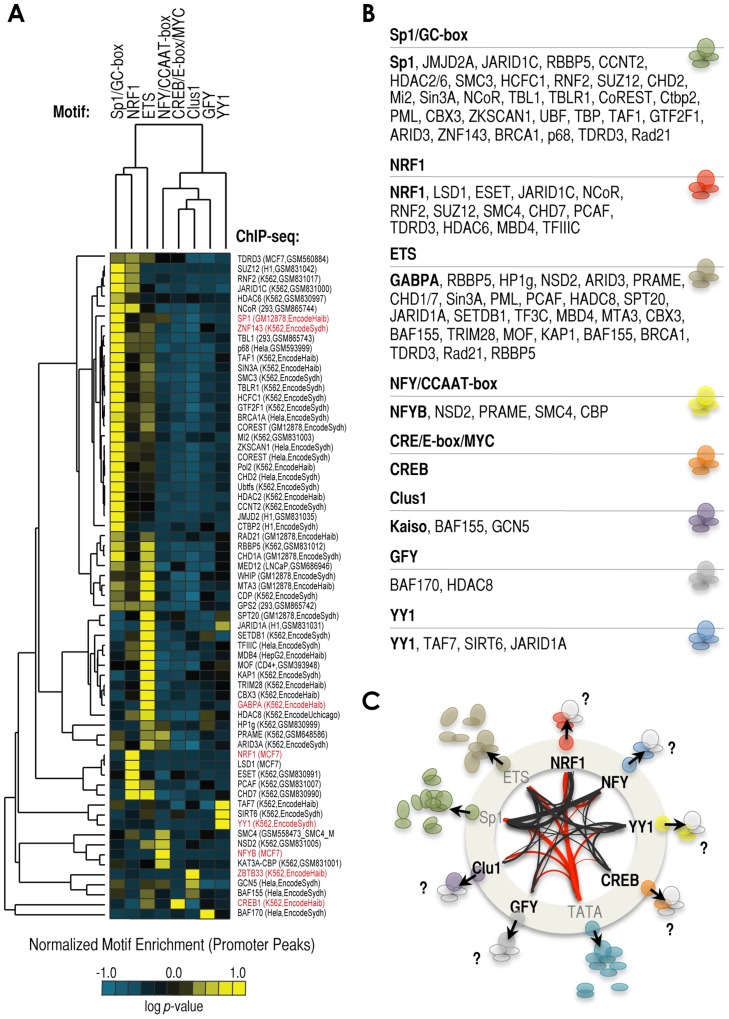
Signature of cofactors associated with the enrichment of a specific cardinal motif. *(* ***A*** *)* Heatmap analysis of relative enrichment of cardinal motifs in proximal promoter regions occupied by different proteins, based on >60 ChIP-seq datasets: n = 8 TF ChIP-seq datasets (labeled in red) and n = 59 cofactors/others ChIP-seq datasets (labeled in black). Sources of ChIP-seq experiments: ENCODE (accession number and/or laboratory are included in parenthesis); and NRF1, NFYB, and LSD1 datasets were generated in this study. The vector of motif enrichment for each experiment was normalized and centered on the mean value to reveal the preferences of each experiment for cardinal motifs. The analysis shows negative log of the hypergeometric *p*-value. *(* ***B*** *)* List of cofactors associated with each cardinal motif, based on (*A*). *(* ***C*** *)* Model of signatures of cofactors associated with the presence of a specific cardinal motif (see text for details).

Finally, we explored the existence of additional strong partnerships associated with cardinal motifs. We analyzed a number of ENCODE ChIP-seq experiments and data from multiple sources to generate a heatmap of motif/cofactor preferences at promoter regions (**Figure 5A**). As predicted by our model, the set of TFs binding to the most highly enriched cardinal elements show no significant preference for motifs other than their cognate sites, thus suggesting that they bind with preference independently and define subsets of promoters (**Figure 5A**: Sp1 as cognate of GC-rich, GABPA as cognate of ETS, and ZBTB33 as cognate of Clus1). Also as predicted by our model, we observed that some cofactors show strong preferences for single or only a few motifs, thus supporting the idea of cardinal motif-associated cofactor signatures (**Figure 5A and 5B**). For example, NRF1 motif-enriched promoters were distinctly associated with LSD1, but also with JARID1C/KDM5C, which is consistent with the result that *KDM5C* siRNA treatment altered the expression of the 3×NRF1-luciferase construct in our screen (**Figure 2B**). We also observed evidence for the preferential binding to NRF1 sites by the histone/protein-methyltranferase ESET, which has been reported to add methyl marks that can later be specifically removed by LSD1 [53], [61], thus suggesting a particular signature of NRF1-LSD1-ESET. Other potential partnerships with NRF1 are: the histone acetyltransferase, PCAF, the ATP-chromatin remodeler, CHD7, the methyl-DNA binding protein, MBD4, the histone/protein deacetylase (HDAC), HDAC8, the E3 ubiquitin-protein ligase, RING2, corepressors SUZ12 and NCoR, and the dimethyl arginine binding protein, TDRD3 (**Figure 5B**). Some of these cofactors were distinctly associated with NRF1 sites (for example, LSD1 and ESET), while others were associated with other motifs (for example, CHD7 and NCoR; **Figure 5A**). Perhaps, it was initially expected that those motifs with higher number of associated cofactors were Sp1/GC-rich and ETS motifs, because these are two of the top-most enriched cardinal sequences (**Figure 1A**). However, using the same argument, it was surprising to observe that few number of cofactors were associated with NRF1, NFY, and CREB/E-box/MYC motifs (**Figure 5A**), considering that their enrichment in human promoters is comparable at least to that of ETS motifs (**Figure 1A**). Overall, these data reveal that cardinal elements may define strong cofactor signatures.

To also test the model of cardinal motif-cofactor regulatory signatures experimentally, we engineered nine constructs to contain NRF1 and/or NFYB motifs in the context of the sequence of their ‘natural’ proximal promoter (−150/+50 bp), but upstream the luciferase gene, and determined the effect on this gene of depleting the same 27 KDMs that we tested in our original screen with multimerized sites (**Figure 2B**). Three of these engineered regions are targets of NRF1, but not NFYB; three are targets of NFYB, but not NRF1; and three are targets of both (based on ChIP-seq data, **Figure S8A**). According to our model, they should show at least two main (or perhaps three, adding the case of mixed NRF1-NFY promoters) basic patterns of KDM siRNA-mediated effects as result of their cardinal NRF1 or NFY motif composition. In fact, we observed that the global effects of the KDM siRNA treatments on the three NRF1-driven promoters clustered together, while the global effects of the same treatments on the three NFY-driven promoters did the same (**Figure S8B**). Analyses of the mixed promoters (whose alignment to the proposed model was initially harder to predict) resulted in two of the promoters clearly clustering with NRF1-driven promoters, while the third promoter still clustered with them, but less clearly (**Figure S8C**). Thus these results (based on a limited test) suggest that cofactor signatures at promoters that contain a combination of cardinal motifs resemble those of promoters with single cardinal motifs, instead of representing a mix of effects induced by the individual motifs, or showing a completely new regulatory signature. More in general, this test with nine constructs reinforces our proposed model that cardinal motifs are strong determinants of cofactor signatures at proximal promoter regions.

## Discussion

For decades, promoters have been known to be critically important in gene transcription regulation [5], [6], [14]. Recently, new approaches that allow analyses of chromatin structure at genome-wide scale are adding new and more global perspectives to our understanding of these regions. For example, these analyses reveal that most human promoters are ‘open’ chromatin regions that center between −150 and +50 bp relative to the TSS, although spread less intensively in slightly wider regions [2], [9]. This finding suggests that the bulk of TF binding and cofactor activities occurring at promoters might be limited to these short genomic regions. Consistent with this possibility, a recent functional analysis of 46 promoters indicated that regions immediately next to the TSS are necessary but also sufficient to control basal transcription [62]. In an effort to understand how these short regions operate on a genome-wide scale, and more particularly to understand their mechanisms of cofactor recruitment, we have explored the role of cis-regulatory elements that are highly enriched at −150/+50 bp in dictating the recruitment of cofactors, in order to establish some basic rules. We have termed these elements as cardinal motifs. Our studies suggest that cardinal motifs tend to occupy −150/+50 bp regions independently rather than in fixed combinations, and that they direct signatures of cofactor recruitment that allow us to classify human promoters into subgroups based on these motifs and their cofactor signatures (**Figure 5C**). The set of cardinal elements that we have identified in our analysis of n = 21,000 human promoters is not substantially different than that uncovered in previous computational analyses [37], [38], [42]–[45]. Similarly, the poor co-occurrence among these motifs in proximal promoter regions was suggested in the past [37] (we show a direct comparison between our analysis and this previous study in **Figure S9A**). Perhaps the major differences between this report and this study are quantitative in terms of motif co-occurrences (**Figure S9A**). With respect to the few qualitative differences, this previous study identified an additional motif, USF, which we did not find in our analysis; in contrast, this previous study did not include the YY1 and GFY elements, which we observe as highly enriched in our dataset (**Figure S9A**). These differences might be explained in part by the facts that we used a different algorithm and that analyzed a different number of promoters and length of sequences in our tests (we studied n = 21,000 human promoters between −150 bp and +50 bp relative to TSS, compared to n = 13,010 human promoters between −1,000 bp and +500 bp in the previous study). In any case, the model and its functional implications were not experimentally tested in this previous study, which is what we attempt here.

In a way, the model that cardinal motifs (or TFs) prefer acting independently than in fixed combinations contrasts with the observation that motifs (or TFs) often operate as combinations at distal ‘open’ chromatin regions (i.e. enhancers and other genomic elements; e.g. [20]–[23], [63]). Therefore, the *dominance* of a single cardinal motif at a single −150/+50 bp region might be a feature rather ‘exclusive’ of ‘open’ chromatin regions at promoters. Our model, however, does not exclude the possibility that cardinal motifs (via the TFs that recognize them) may act combinatorially via short- or long-range interactions in the nuclear space, or in *trans*, with other motifs (or TFs). It does not contradict either previous reports indicating that cardinal motifs act synergistically or cooperatively in many proximal promoter regions (e.g. [64]–[68]). In fact, we may see this phenomenon in a significant number of cases (e.g. see examples in **Figures 3A** **and S7**, and especially **Figure S2C** and **S2D**). But we propose that these cases do not represent the most general rule (**Figures 1E** **and S2C**). Furthermore, we tested whether the preference to occupy different promoter subsets is maintained if the margins of the promoter sequence for analysis are wider (**Figure S2A**) or are defined experimentally based on DHSs (**Figure S1B**), achieving the same conclusion. We also tested whether this preference is maintained when proximal promoters are subclassified based on the mode of transcription initiation (‘focused’ or ‘dispersed’ [5], [6]). In particular, we took advantage of available 5′ RNA-seq data and identified 2,838 focused and 5,220 dispersed promoters in MCF7 cells (see Methods for details). As expected, focused promoters show higher preference to contain TATA and YY1 motifs (blue, **Figure S9B**). Dispersed promoters show higher preference to contain ETS and NRF1 motifs (red, **Figure S9B**). Despite these preferences, the observed co-occurrences between TATA and YY1 or ETS and NRF1 are not enriched within each group (**Figure S9C**).

What is the role of cardinal elements at proximal promoters? Since −150/+50 bp regions are at the center of promoter DHSs [2], it is an interesting possibility that TFs recognizing these motifs are responsible of the typical chromatin features of these regions (‘open’ chromatin surrounded by nucleosomes that are heavily modified). At enhancers, a special class of TFs termed ‘pioneer factors’ have been suggested to contribute to their chromatin organization [69]. Pioneer factors can be distinguished by their ability to bind first in a temporal sequence of additional binding events, and to bind independently rather than cooperatively to chromatin [69]. Pioneer factors can also be distinguished since they establish competence for gene expression, rather than activate transcription [69]. Examples of pioneer factors include: lineage-specific transcription factors, FoxA1/3 and GATA3/4. The fact that cardinal TFs are often constitutively bound to the genome, as pioneer factors FoxA1/3 and GATA3/4 at enhancers, and that some of them remodel chromatin [70], makes them qualify as candidate pioneer factors. If, furthermore, we consider our model that cardinal TFs prefer binding independently to many promoters, it would be a third property of pioneer factors. Thus TFs recognizing cardinal motifs might be in fact ‘promoter-specific pioneer factors’. However, we can only speculate on this possibility, since other typical features of a pioneer factor, such as be permissive for transcription rather than directly activate transcription, and bind first in a sequence of binding events, are not obvious in the case of cardinal TFs.

Based on: 1) our analysis of >60 ChIP-seq experiments revealing that each cardinal motif might be associated with the selective recruitment of a subset of cofactors (**Figure 5A**); 2) the observation that knockdown analysis of a large panel of KDMs shows that proximal promoters regulated by the same motif exhibit relatively similar patterns of regulation (**Figure S8**); 3) that three copies of a single motif could dictate a complex regulatory pattern of KDMs (**Figure 2B**); and, 4) that some cardinal TFs strongly associate (biochemically and functionally) with specific cofactors (e.g. NRF1 and LSD1); we propose here that a main role of cardinal elements at proximal promoters is to dictate a signature of cofactors. What is the functional relevance of dictating these signatures? These cofactors may potentially be involved in dictating the particular chromatin structure of these regions, and/or control of transcription initiation and RNA PolII pause-release. If that is the case, it is intriguing that two of these elements (Sp1/GC-rich and ETS) account for most of the cofactor binding preferences that we have identified in our analysis (see **Figure 5B**). Initially, this observation may suggest that there are a series of promoter subclasses of which we might not know much yet about their regulation. However, it is also possible that our analysis of cofactors was too restricted (limited to around 60 ChIP-seq datasets), thus we may have specifically missed cofactors that are associated with other cardinal motifs. It is also possible that ChIP-seq data for those cofactors that are specifically associated with cardinal motifs other than Sp1/GC-rich and ETS are not yet available in the literature. For example, PGC1α is a cell type-specific cofactor that is well known to regulate NRF1-dependent transcription, but we did not find available ChIP-seq data for this specific cofactor. But it is also possible that there is no bias in our analyses or in the list of cofactors profiled up-to-date by ChIP-seq, and that in fact there are two basic regulatory strategies for the way cardinal motifs regulate transcription. For example, some elements (such as Sp1/GC-rich and ETS) might be associated with heavier promoter-dependent regulation, while other elements (such as NRF1, NFY/CCAAT, CREB/MYC, and YY1) might be subject to heavier enhancer/distal-dependent regulation, thus revealing a genuine and essential regulatory difference between these two groups. In fact, CBP is a well-known cofactor of CREB, but CBP preferentially binds to distal sites, in contrast to CREB, which preferentially binds to promoter regions [39], perhaps sin agreement with this possibility. Further studies will be necessary to elucidate a broader and clearer picture of the role of cardinal elements in selective recruitment of cofactors.

Based on the relatively low number of cofactors we currently know that act via cardinal motifs other than Sp1/GC-rich or ETS (**Figure 5B and 5C**), our finding that the subset of promoters *dominated* by NRF1 elements is strongly associated with LSD1 is of special importance. LSD1 (also known as KDM1A, AOF2, and BHC110) was the first KDM discovered [71]. LSD1 is a flavin adenine dinucleotide (FAD)-dependent amine oxidase, which requires FAD, an intermediate metabolite of the mitochondrial respiratory chain, to remove methyl marks from histone and non-histone proteins [71]–[73]. Interestingly, we have observed that the ‘NRF1/LSD1’ signature is associated with the control of nuclear-encoded mitochondrial genes, which are also well-known NRF1 targets (**Figures S5C, S5D** and **S6**). Furthermore, genes in which LSD1-acts as a coactivator of NRF1 (*Class I* in **Figures 4D and 4E**) show “mitochondrial” and “RNA processing” functions as the most significantly associated GO terms (**Figure S10A**). Although similar functions were also associated by GO analysis of genes regulated (functionally) independently by NRF1 and LSD1 (*Class V* and *VII*; **Figures 4D, 4F** **, S10C**, and **S10E**). In fact, it is remarkable that LSD1 occupies an impressive 1/3 of all active, nuclear-encoded mitochondrial genes in MCF7 cells (considering only those promoters that are H3K4me3-positive or active in these cells; **Figure S10G**). Similarly, an analysis of protein-protein interacting networks of LSD1 peaks suggests that LSD1 regulates “mitochondrial metabolism”, as well as “RNA metabolism/translation” and “cell cycle” (**Figure S11**). Previous studies already associated LSD1 to mitochondrial functions in fission yeast (*S. pombe*) [74]. Moreover, it has been reported that the levels of FAD modulate the switch to lipid storage in mouse adipocytes via repressing *PGC1β*/*PPARGC1B*, *PDK4*, *FATP1/SLC27A1*, and *ATGL/PNPLA2* genes in an LSD1-dependent manner [75]. However, we did not find LSD1 at the promoters of these four specific targets in MCF7 cells, and these genes were not expressed in these cells (data not shown), perhaps suggesting an adipocyte-specific, LSD1-dependent regulatory mechanism. In fact, mitochondrial regulation is not identical in every cell type, and the content and morphology of mitochondria are largely determined by nuclear-encoded genes of variable expression across tissues [76], [77]. In our analyses, LSD1 may also act as NRF1 negative modulator (*Class VI*; **Figures 4D and 4F**), but this repressive activity is associated with cell motility, signaling, and cell adhesion, among other GO terms (**Figures S10D**). In summary, the pervasive association between NRF1 and LSD1 cannot be interpreted as associated with a single or universal functional outcome, although when acting as NRF1 coactivator seems strongly associated with control of mitochondrial metabolism and biogenesis (**Figures S12**).

In MCF7 cells, we found that 76% of LSD1 peaks occur at or near H3K4me3-marked regions, which corresponded to epigenetically defined promoter regions (**Figure S4C**). This result is in agreement with the finding that LSD1 is a component of the >2MDa MLL1 complex [78], since MLL1 can also be found associated to thousands of promoters in the human genome [79]. However, LSD1 does not seem to act dominantly via promoters in every cell, which might be expected since LSD1 interacts with many TFs in a cell-type-specific manner [53]. For example, the binding map of LSD1 in erythromyeloblastoid leukemia K562 cells [3], [30] only marginally overlaps with that in MCF7 cells (3% at −150/+50 bp), since most LSD1 binding in K562 cells is distal. It is unclear to us why LSD1 binds strongly to promoters in MCF7 cells and other cell lines [53], [80]–[82] (this can also be observed using the datasets of others [54], [83]), while in K562 cells it shows poor association with promoter regions. We suspect that some cells represent special cases, but this demands further exploration.

In conclusion, we propose a general model in which cardinal cis-regulatory elements acting via promoter DHSs dictate the selective recruitment of cofactors to specific promoters, thus establishing a co-regulatory code that would be largely distinctive of each cardinal motif and promoter subset defined by these elements. We have started to decode this signature-based model, but it will be necessary to identify the full repertoire of ‘regulatory logic operations’ (as first defined in [84]) to have a complete understanding of how these motifs function.

## Materials and Methods

### Cell culture and antibodies

Human breast cancer MCF7 cells were cultured in DMEM(1×)+GlutaMAX-I medium (Life Technologies) supplemented with 10% fetal bovine serum (FBS, Omega Scientific). Human prostate cancer LNCaP cells were cultured in Advanced DMEM/F12(1×) medium (Life Technologies) supplemented with 10% FBS. Human osteosarcoma U2OS cells, human embryonic kidney (HEK) 293T cells, and human neuroblastoma SH-SY5Y cells were cultured according to The American Type Culture Collection (ATCC) protocols. Primary normal human epithelial cells (HMEC, CC-2651) were cultured using media and protocols provided by Lonza Bioproducts, commercial supplier of these cells. Cell lines were maintained in cell incubators at 37°C and 5% CO_2_. MCF7, LNCaP, and HMEC cells were hormone-deprived for 4 days in phenol-free plus charcoal-depleted FBS before each experiment, and then treated 1 hr with 100 nM 17β-estradiol (E2, Sigma-Aldrich) in the case of MCF7 and HMEC cells, or with 100 nM dihydrotestosterone (DHT, Sigma-Aldrich) in the case of LNCaP cells, as previously reported [22], [50], [53]. Anti-LSD1 antibodies were previously described [53]. Anti-NRF1 (PAC102) antibodies were a generous gift from Dr. Danny Reines [64]. Anti-NFYB (H-209, sc-10779), anti-FoxA1 (C-20, sc-6553), anti-RNA PolII (N-20, sc-899), and anti-ERα (HC-20, sc-543) antibodies were purchased from Santa Cruz Biotechnology. Anti-H3K4me3 (07-473) antibodies were purchased from Upstate/Millipore. Anti-actin (MAB1501) antibodies were purchased from Chemicon.

### Source of promoter sequences and publically available datasets

Core promoter sequences from −150 to +50 bp relative to TSS were extracted from the UCSC genome browser by using genome assembly hg16 and mm3. 5′ RNA-seq data from MCF7 cells is available at the Database of Transcription Start Sites (DBTSS). The following were the sources of DNaseI-seq, MNase-seq, ChIP-seq, and ChIP-DSL data generated in previous studies and used here: RNA PolII ChIP-seq in E2-treated MCF7 cells reported in [85]; RNA PolII ChIP-DSL in E2-treated MCF7 cells reported in [50]; ERα ChIP-seq in E2-treated MCF7 cells reported in [86]; DNaseI-seq and H3K4me2-MNase-seq in MCF7 cells reported in [87]; LSD1 ChIP-DSL in E2-treated MCF7 cells reported in [53]; and LSD1 ChIP-seq in ESCs reported in [54]. Multiple datasets were also generated by The ENCODE Project (as indicated in figure legends). The rest of ChIP-seq and ChIP-DSL experiments are reported in this study.

### Identification and visualization of enriched cis-regulatory elements

De novo motif discovery analysis was performed by HOMER (http://biowhat.ucsd.edu/homer/), as described in previous studies [22], [23], [27], [50]. Motif enrichment at these regions was determined in comparison to background regions randomly selected from the genome matched for GC%. Sequence logos were generated using the web-based application, WebLOGO (http://weblogo.berkeley.edu). Motif enrichment heatmaps and dendrograms were created by clustering Hypergeometric log *P*-values using open source software Cluster (http://bonsai.ims.u-tokyo.ac.jp/~mdehoon/software/cluster/software.htm#ctv).

### Motif enrichment preference in cofactor ChIP-seq datasets

For enrichment analysis of cardinal motifs in publically available cofactor ChIP-Seq datasets, we first identified promoter regions containing ChIP-Seq-identified cofactor peaks within 500 bp of the annotated TSS (we used peak lists provided by the authors reporting each ChIP-seq dataset). We determined the enrichment of each cardinal motif by HOMER in each set of promoters (negative log of the hypergeometric *p*-value). The vector of motif enrichment for each experiment was then normalized and centered on the mean value to reveal the preferences of each experiment for cardinal motifs.

### Motif enrichment preference in focused and dispersed promoters

To determine motif enrichment preferences of cardinal motifs in promoters subclassified based on their mode of transcription initiation, we took advantage of publically available 5′ RNA-Seq data obtained in MCF7 cells deposited in DBTSS. We defined focused and dispersed promoters as having, respectively, ≥90% and <90% 5′ RNA reads within ±5 bp of the primary TSS relative to the surrounding 100 bp promoter. This criteria identified n = 2,838 (35%) focused and n = 5,220 (65%) dispersed promoters in these cells. We then analyzed the preference of each group to contain a specific cardinal cis-regulatory element by HOMER.

### ChIP and ChIP-seq assays

Chromatin immunoprecipitations (ChIPs) for standard RT-qPCR analysis and ChIP-seq/DSL experiments were performed as previously described [22], [50], [53]. RT-qPCRs for standard ChIPs were conducted in an Mx3000P Real-Time PCR Instrument (Agilent) with 2×Brilliant qPCR Master mix (Stratagene). PCR settings were the following: 10 min at 95°C followed by 40 cycles of 95°C for 15 sec, 58°C for 15 sec, and 25 sec for 72°C. Primer sequences were the following: *FMR1-*forward 5′-CCAGGCCACTTGAAGAGAGA-3′ and *FMR1-*reverse 5′-TGCGGGTGTAAACACTGAAAC-3′; *TFAM-*forward 5′-ACCGGATGTTAGCAGATTTCC-3′ and *TFAM-*reverse 5′-CCTCCTGGCAATACACAACTC-3′; *FXR2-*forward 5′-CAAGGTTAGAGCCCCAGCTA-3′ and *FXR2-*reverse 5′-GCGGTGAAGAAAGAAGGCTA-3′; *GAPD-*forward 5′-TCCTCCTGTTTCATCCAAGC-3′ and *GAPD-*reverse 5′-TAGTAGCCGGGCCCTACTTT-3′; and, *UMODL1-*forward 5′-CCTTCAGTTCCCGGGAGTA-3′ and *UMODL1-*reverse 5′-CTGGAAGGAAATACGTCCACA-3′. For ChIPs in plasmids, we engineered a construct with 3×NRF1 sites upstream the minimal promoter in the pGL2 plasmid (see ‘siRNA screen’ for more details about this construct, below) and a mutant 3×NRF1 version that contains the following fragment: 5′-TGTTTATTTTCAGacgtacgtTGTTTATTTTCAGacgtacgtTGTTTATTTTCAG-3′. Chromatin for ChIP to test plasmids was prepared as for standard ChIPs. Analysis of ChIP-seq experiments was previously described [22], [23], [27]. ChIP-seq experiments were performed in an Illumina GA2 sequencing instrument. DNA libraries were performed as previously described [22], [27]. Biological triplicates (n = 3) were pooled before DNA library preparation. A full list of NRF1, NFYB, and LSD1 ChIP-seq peaks can be found in **Supplementary Table S1, S2, S3.**

### ChIP –DSL assay

ChIPs followed by DSL (DNA, Selection, and Ligation, or ChIP-DSL) were performed and analyzed as previously described [50]. Briefly, the ChIP-DSL assay uses ChIP DNA as a template for oligonucleotide ligation, not for direct amplification. ChIP-DSL is based on DNA-mediated isolation (or selection) of a pool of pre-designed 40-mer oligonucleotides (plus T3 and T7 5′ ends) that are synthesized as pairs (T3+20-mer and T7+20-mer) but that can be easily ligated once associated to their correct DNA template in the genome, since both should anneal adjacently. Ligated oligonucleotides can be then amplified after release from the template based on the presence of T3 and T7 sequences. These oligonucleotides are carefully pre-designed to anneal at similar temperature and having similar GC content. In a typical ChIP-DSL experiment, ChIP'ed DNA is purified and then randomly biotinylated. Biotinylated DNA is incubated with the pool of DSL (T3+20-mer and T7+20-mer) oligonucleotides before isolation with streptavidine-coated beads. After extensive washing of excess (not annealed) oligonucleotides, those annealed in to their right DNA template can be ligated based on their close proximity. After amplification, PCR fragments (all the same size, with similar annealing temperature, and similar GC content) are hybridized to an array of complementary sequences. We performed ChIP-DSL experiments in triplicate (n = 3). For LSD1, NRF1 and NFYB ChIP-DSL analyses, we hybridized the samples onto the Hu20K array, which contains 40-mer sequences for n≈20,000 human proximal promoters between −800 bp and +200 bp (one sequence per promoter). The experimentally calculated false positive rate for a PolII ChIP-DSL experiment in MCF7 cells is 3%, and the false negative rate is 33% in the Hu20K array [50]. For Sp1 ChIP-DSL analysis, we hybridized the samples onto the Hu2K array, which is a small version of the Hu20K array with mostly promoters of cell cycle-regulated genes. As an additional note, the numbers and percentages provided in the different panels/figures with ChIP-DSL experiments were calculated based on the actual number of spots on the array providing reliable signal (e.g. n = 17,288 in the Hu20K array), not for the total number of promoters actually spotted on the array (n = 20,000 in the Hu20K array). A full summary of ChIP-DSL positive hits can be found in **Supplementary Table S4.**

### siRNA/plasmid transient transfection

All transient transfections (siRNAs and plasmids) were performed with Lipofectamine 2000 following the manufacturer's protocol (Invitrogen). Transfections were performed one day after seeding of cells, and the effects induced by the siRNA treatments were measured after 2/3 days. Transient transfections in HEK293T and U2OS cells were performed at 70–90% confluency. Transient transfections in MCF7 cells were performed at 10–20% confluency. Specifically for the siRNA screen with multimerized 3×NRF1 or 3×NFY sites and for the gene expression analyses based on RT-qPCR and microarrays, transient transfections were performed in 6-well plates. For the siRNA screen with natural promoters and for transient transfections, experiments were performed in 96-well plates. In 96-well plates, we transfected 8.67 pmols of siRNA (purchased from Sigma-Aldrich), 0.1 µg of pGL2-Luciferase constructs (see below), and 0.01 µg pRL(Renilla)-TK.

### siRNA Screen and dual Luciferase/Renilla assay

We measured dual Luciferase/Renilla expression with the Dual-Glo Luciferase Kit (Promega) using a Veritas Microplate Luminometer (Turner Biosystems/Promega) and following standard procedures. Collected data were normalized (Firefly signal/Renilla signal) and referred to the control (scrambled siRNA) value. We engineered two constructs for the siRNA screen with multimerized sites containing 3×NRF1 or 3×NFY/CCAAT sites upstream the luciferase gene in the pGL2 plasmid harboring the TK minimal promoter. The two cloned fragments were the following: 5′-TGCGCATGCGCAGacgtacgtTGCGCATGCGCAGacgtacgtTGCGCATGCGCAG-3′, which contains three copies of the NRF1 site found in the *FMR1* promoter (in uppercase) [64]; and tctgATTGGctggttaaggcatctgcttaacttctgATTGGctggttaaggcatctgcttaactacgATTGGcta, which contains three copies of a consensus NFY/CCAAT-box. For the siRNA screen with sites in the context of their natural promoters, we engineered the following regions upstream the luciferase gene in the pGL2-basic plasmid: −153/+29 bp from the *GDPD1* promoter, −158/+30 bp from the *ASNSD1* promoter, −150/+50 bp from the *ZBTB17* promoter, −150/+50 bp from the *ZNF695* promoter, −150/+50 bp from the *STIP1* promoter, −150/+50 bp from the *AKAP8L* promoter, −150/+37 bp from the *ZWINT* promoter, −400/+50 bp) from the *RFX1* promoter, and −150/+100 bp from the *CCT3* promoter. These regions and their margins were selected based on our NRF1 and NFYB ChIP-seq datasets, and contain NRF1 peaks and recognizable NRF1 motifs (for *GDPD1*, *ASNSD1*, *ZBTB17*), NFYB peaks and recognizable NFY/CCAAT motifs (for *ZWINT*, *RFX1*, and *CCT3*), or NRF1 and NFYB peaks and recognizable NRF1 and NFY/CCAAT motifs (for *ZNF695*, *STIP1*, and *AKAP8L*). The position of the TSS was based on the gene annotation in the UCSC browser. For data visualization of the siRNA screen results with natural promoters, we represented normalized luciferase signal in heatmaps created by clustering Hypergeometric log values using Euclidean distance and average linkage with TMEV 4.9 Software (Dana-Farber Cancer Institute).

### RNA isolation, RT-PCR, and RNA profiling analysis

Total RNA was extracted with the RNeasy Kit (Qiagen) and DNA was eliminated with on-colum DNase treatment (Qiagen) following the manufacturer's protocols. Total RNA was converted into cDNA with the SuperScript First-Strand Synthesis Kit (Invitrogen) following the manufacturer's protocol. Real-time PCR was conducted in a Mx3000P Real-Time PCR Instrument (Agilent). Brilliant qPCR Master mix (Stratagene). PCR settings were the following: 10 min at 95°C followed by 40 cycles of 95°C for 15 sec, 58°C for 15 sec, and 25 sec for 72°C. Primer sequences were the following: *TFAM*-forward 5′-GTGATTCACCGCAGGAAAAG-3′ and *TFAM*-reverse 5′-CTGGTTTCCTGTGCCTATCC-3′, *FXR2*-forward 5′-AACCGTGGTAATCGGACTGA-3′ and *FXR2*-reverse 5′-GGTGCAGGTTGGAGGTTTTA-3′, for *CDC42*-forward 5′-TACTGCAGGGCAAGAGGATT-3′ and *CDC42*-reverse 5′-CCCAACAAGCAAGAAAGGAG-3′, *CDC2*-forward 5′-CCATGGGGATTCAGAAATTG-3′ and *CDC2*-reverse 5′-CCATTTTGCCAGAAATTCGT-3′, *SAP18*-forward 5′-TGGATGCAACCTTGAAAGAA-3′ and *SAP18*-reverse 5′-TGGAATCATCAGTCCCCTTT-3′, and *ACTB*-forward 5′-GTGGGCATGGGTCAGAAG-3′ and *ACTB*-reverse 5′-TCCATCACGATGCCAGTG-3′. Cycle threshold (Ct) values were extracted with MxPro qPCR Software (Agilent) to calculate difference Ct (ΔCt) values with respect to control. For microarray analysis, cDNA quality was assessed by the Agilent Bioanalyzer (Agilent). Gene expression profiling was performed using the Human Illumina Sentrix Expression BeadChips system, as previously described [50] in the Biogem core (UCSD). Genome expression data are available in the GEO database.

### Co-immunoprecipitation assay

Co-immunoprecipitation (co-IP) experiments were carried out as previously described [88]. Briefly, three 10 cm plates of confluent MCF7 cells were washed with ice-cold PBS. Cells were then disrupted and homogenized with IPH buffer (50 mM Tris-HCl, pH 8.0, 150 mM NaCl, 5 mM EDTA, 0.5% NP-40, with Roche's cocktail protease inhibitors). Samples were incubated on ice for 20 min and cleared by maximum centrifugation (14,000×g) for 10 minutes at 4°C. The supernatant volume was divided into four aliquots for overnight incubation with specific antibodies. Immuno-complexes were isolated after 2 hrs incubation with protein A beads (Sigma-Aldrich). Protein A beads were then pelleted and washed 3 times in IPH buffer. Protein A beads were then resuspended in loading buffer and the solution was analyzed by Western blot.

### Size exclusion chromatography

Nuclear extracts from MCF7 cells were prepared fresh similarly as in [89], with the following modification: nuclear pellet (after discarding cytoplasmic and membranous fractions) was carefully resuspended in nuclear extraction buffer (20 mM HEPES-KOH pH 7.9, 25% glycerol, 400 mM NaCl, 1.5 mM MgCl2, 0.2 mM EDTA, 0.5 dithiothreitol, 0.05% NP-40, and Roche's cocktail protease inhibitors) and maintained on ice for 40 min. The soluble fraction was obtained after centrifugation for 10 min at 14,000×g and 4°C. This fraction was then loaded onto a Superose 6 column (Pharmacia) previously equilibrated with nuclear extraction buffer without glycerol. The column was applied into the FPLC system and ran following manufacturer's recommendations (Pharmacia), as previously reported [90]. Elution fractions of 0.5 mL were collected and analyzed by Western blot. Previously, we ran an independent sample of proteins of known molecular sizes (HMW or high-molecular weight Calibration kit, Pharmacia) to determine elution volumes based on protein size and the size exclusion (void) of the column. This set of proteins markers contained the following: blue dextran (>2MDa), thyroglobulin (669KDa), ferritin (440KDa), and catalase (232KDa).

### Western blotting

Proteins were loaded and run on 4–12% Bis-Tris gels with MES running buffer (Life Technologies). After transfer onto 0.2 µm-pore PVDF (BioRad) or nitrocellulose (Whatman) membranes, membranes were blocked with 5% milk/TBST for 30 min and probed with antibodies diluted in 5% BSA/TBST overnight at 4°C. Immunodetection was achieved after incubation with HRP-conjugated (Invitrogen) goat anti-mouse or goat anti-rabbit diluted 1∶5,000 in blocking solution. HRP signal was detected by ECL (Amersham-GE) and autoradiography film.

### Functional gene annotation analysis

The analysis of functional gene annotations was performed using the Database for Annotation, Visualization, and Integrated Discovery (DAVID) 2.1 website [91]. We queried functional annotations using official gene symbols. We selected genes for analysis based on three different criteria: genes in which we detected NRF1 or NFYB peaks at their −150/+50 bp regions relative to TSS based on ChIP-seq data; genes in which we detected NRF1 or NFYB binding at their −800/+200 bp regions (*p*<0.0001) based on ChIP-DSL data; and genes affected by different siRNA treatments (specified in figure panels) based on gene expression microarrays. A representative selection of the most enriched functional terms and the *p*-value of their enrichment were extracted for visualization using Excel.

### Network analysis

Networks of annotated biological functions associated with LSD1 targets were constructed with open source Cytoscape version 2.8.3 [92], and the following plugins: MiMI, which integrates data from multiple databases [93]; MCODE, which finds highly interconnected regions in a (sub)network [94]; NetworkAnalyzer, which computes and displays networks [95]; and Random Network, which generates random networks and compare them to existing networks. We searched the following databases: BIND, CCCB, DIP, GRID, HPRD, IntAct, KEGG, MDC, MINT, PubMed, and Reactome. For visualization, we show only interactions between query genes. List of network attributes: gene name (closest gene to a LSD1 ChIP-seq peak), Entrez gene ID, absolute distance from TSS (bases), peak genomic localization annotation (promoter, intron, exon, TTS, intergenic), and ChIP-seq signal (Tag counts). List of visualization parameters: ‘node color’ represents proximity to TSS (green gradient, <400 bp; white, at 400 bp; red gradient, >400 bp); ‘node size’ represents ChIP-seq signal intensity; ‘node shape’ represents genomic location annotation (promoter = circle; exon = rectangle; intron = diamond; TTS = triangle; and intergenic = hexagon). Sub-networks were generated with MCODE and those with the highest score were selected. Network statistics were calculated using NetworkAnalyzer plugin, which provides clustering coefficients for (sub)networks, and Random Network plugin, which provides clustering coefficients for random (sub)networks. We applied paired two-tailed t-test to compare (sub)network with identical (same number of nodes) randomly generated (sub)networks.

### Statistical analysis

Non-sequencing experiments were performed in triplicate (including ChIP-DSL experiments), while sequencing experiments were performed in triplicate but pooled before DNA library preparation. Data is presented as mean ± standard error of the mean (s.e.m.) of replicates. Comparisons between two groups were performed using paired two-tailed t-test. *P*-values<0.05 were considered statistically significant. Additional information about statistical analyses is provided in independent subsection (see above).

## Supporting Information

Figure S1 Cardinal motifs tend not to co-occur. (***A***) A summary (manually drawn with PowerPoint) of positive (left) and negative (right) motif co-occurrences identified in −150/+50 bp regions, based on **Figure 1B**. Connecting lines indicate a positive (black) or negative (red) co-occurrence between two cardinal motifs, and line thicknesses approximately correlate with levels of co-occurrence. (***B***) Co-occurrence matrix of cardinal motifs in n = 10,063 DHS regions defined experimentally by DNaseI-seq in human MCF7 cells that overlapped with RefSeq promoters. Co-occurrence log_2_ *p*-values are shown as a gradient of blue-to-red for positive-to-negative co-occurrence, and as white for no significant co-occurrence. (***C***) Distribution of NRF1 (left) and NFYB (right) ChIP-seq peaks with respect to the genome annotation. The numbers included in the pie charts refer to the fraction of peaks associated with each annotated region. The total number of peaks analyzed is also indicated. (***D***) Distribution of sequencing read density, based on H3K4me3 ChIP-seq, around NRF1 and NFYB ChIP-seq peaks (centre of the panel) in MCF7 cells. (***E***) Distribution of computationally predicted cardinal motif densities (black) and sequencing read density of: TF ChIP-seq data (red), DNaseI-seq data (light blue), H3K4me2 ChIP-seq data (light green), and H3K4me3 ChIP-seq data (dark green) with respect to TSS (vertical line at position 0). On top, the name of the specific motif and TF (in parenthesis) analyzed is shown. Doted vertical lines indicate −150 bp and +50 bp positions. DNaseI-seq, H3K4me2/3 ChIP-seq, NFYB ChIP-seq, GABPa ChIP-seq, NRF1 ChIP-seq, and MAX ChIP-seq data were obtained in MCF7 cells. Sp1, YY1, ZBTB33, and TBP ChIP-seq experiments were obtained in K562 cells. Source of sequencing datasets: all but H3K4me3, NRF1, and NFYB ChIP-seq experiments were produced by ENCODE and are publically available.
(PDF)

Figure S2 Poor rate of colocalization between cardinal TFs NRF1 and NFYB at −150/+50 bp regions and beyond. (***A***) Venn diagram depicting the overlap of NRF1 (red circle), NFYB (blue circle), and RNA PolII (grey circle) in MCF7 cells. NRF1 and NFYB data was based on ChIP-seq and peaks found between −800 bp and +200 bp relative to TSS. We considered as ‘overlap’ the coincidence of NRF1 and NFYB peaks in the same −800/+200 bp region. Also, we considered as ‘overlap’ the coincidence of RNA PolII peaks within ±1 kb of a TSS containing NRF1 or NFYB peaks at −8000/+200 bp. (***B***) Comparison of NRF1 (left) and NFYB (right) ChIP-seq peaks and ChIP-DSL positive hits in MCF7 cells. We compared the lists of −800/+200 bp genomic regions containing NRF1 or NFYB ChIP-seq peaks and the lists of NRF1 or NFYB ChIP-DSL positive hits (or promoters) to determine the number of coincident peaks/hits between both types of analyses. The analysis of ChIP-seq data was limited to the set of genomic regions present on the Hu20K array. We compared three levels of ChIP-DSL stringency based on *p*-values of positive hits: *p*<0.01, *p*<0.001, and *p*<0.0001. The percentage and number of promoters in each case are indicated. (***C***) Venn diagram depicting the overlap of NRF1 (red circle), NFYB (blue circle), and RNA PolII (grey circle) ChIP-DSL positive hits (*p*<0.0001) in MCF7 cells. The ChIP-DSL assay limits the analysis to −800/+200 bp regions relative to TSS. (***D***) Matrix of motif enrichment compared to background of the subset of n = 332 NRF1 and NFYB co-occupied promoters identified by ChIP-DSL. Motif analysis limited to −150/+50 bp regions. Motif enrichments higher than background are shown as a gradient of blue, while motif enrichments lower than background are shown as a gradient of red. No significant enrichment (equivalent to background) is shown as white. (***E***) Functional or GO analysis of genes with promoters occupied by NRF1 (left), NFYB (middle), or NRF1 and NFYB (right) based on ChIP-DSL analysis (*p*<0.0001). Selected GO terms are shown in the y-axis. The *x*-axis refers to *p*-values of enrichment. Orange lines indicate *p* = 0.001.
(PDF)

Figure S3 Strong co-association between cardinal TF NRF1 and cofactor LSD1 at genome-wide scale. (***A***) SiRNA screen based on the luciferase assay to identify KDMs (listed at the bottom) that may act selectively via 3×NRF1 sites (red) or 3×NFY sites (blue). A schematic overview of this screen and a summary of the results are shown in **Figure 2A** and **Figure 2B**, respectively. Selectivity for 3×NRF1 or 3×NFY sites is described in the figure legend of **Figure 2B**. Bottom: *P*-values≤0.05 are shown as grey boxes, *p*-value>0.05 are shown as white boxes. (***B***) Distribution of sequencing read density based on DNaseI-seq (top) and H3K4me2 MNase-seq (H3K4me2-marked nucleosomes, bottom) around LSD1 ChIP-seq peaks in MCF7 cells. (***C***) Venn diagram depicting the overlap of LSD1 (orange), NRF1 (red), and NFYB (blue) ChIP-DSL positive promoters in MCF7 cells based on the Hu20K array (−800/+200 bp). (***D***) Venn diagram depicting the overlap of LSD1 (orange), NRF1 (red), and NFYB (blue) ChIP-seq peaks in MCF7 cells without restriction of genomic localization (whole genome). (***E***) Percentage (top) and relative number (bottom) of LSD1-occupied promoters in NRF1- (red), NFYB- (blue), or Sp1- (green) occupied promoters in MCF7 cells, based on ChIP-DSL data. The percentage (top) and relative number (bottom) of LSD1-occupied promoters in cases that are not occupied by NRF1, NFYB, or Sp1 are also shown (large square). Analysis restricted to the n = 2,000 promoters present on the Hu2K array. The top panel shows percentage of LSD1-occupation relative to the total number of TF-positive promoters. The bottom panel shows absolute numbers of the same analysis. Data for Sp1 ChIP-DSL was obtained using the Hu2K array. Data for LSD1, NRF1, and NFYB ChIP-DSL was obtained using the Hu20K array, but their analyses were restricted to the set of promoters contained in the Hu2K array. Note: the Hu2K array is mostly constituted of cell cycle-regulated promoters, therefore, the percentages/frequencies of occupied promoters by the different TFs/LSD1 in the Hu2K array might be different than those observed in the analysis of the ‘unbiased’ Hu20K array.
(PDF)

Figure S4 Strong co-association between LSD1 and NRF1 at proximal promoters and between LSD1 and ERα at distal sites in MCF7 cells. (***A***) Top-enriched motifs found in LSD1 ChIP-seq peaks in MCF7 cells identified by *de novo* motif discovery analysis. The panel includes: rank of motif enrichment, name of TFs associated with each motif, and *p*-value of enrichment. (***B***) LSD1 binding at genomic regulatory regions of representative examples of well-known ERα-regulated genes. The panel shows ChIP-seq tracks for LSD1 (orange), NRF1 (red), NFYB (blue), ERα (light green), FoxA1 (dark green), and H3K4me3 (black) at ERα-regulated loci: *TFF1*, *CTSD*, and *GREB1*. Refseq annotations are shown at the bottom of each panel. Coincident ERα and LSD1 peaks are enclosed in dotted boxes. (***C***) Heatmap analysis of LSD1, H3K4me3, NRF1, and ERα ChIP-seq signal ±3 kb around LSD1 ChIP-seq peaks (center of the columns). Two sets of LSD1 peaks were separately analyzed: LSD1 peaks nearby H3K4me3-marked regions (i.e. promoters; on top); and, LSD1 peaks nearby H3K4me3-negative regions (likely enhancers; at the bottom).
(PDF)

Figure S5 Strong binding co-association between NRF1 and LSD1 in different cell lines/types. (***A***) Percentage of NRF1 and NFYB occupied promoters that contain computationally predicted NRF1 or NFY motifs (left and right panels, respectively) in MCF7, HMEC, LNCaP, U2OS, and SH-SY5Y cells. Data based on ChIP-DSL analysis. ‘Background’ corresponds to the fraction of approximately 20,000 promoters in the Hu20K array that contain predicted NRF1 or NFY motifs. (***B***) ChIP validation of randomly selected LSD1 target promoters identified by ChIP-DSL in MCF7 cells (top), and ChIP analysis of the same promoters in U2OS cells (bottom). The results are shown as binding fold change over IgG signal. Three promoters were included as negative control (based on ChIP-DSL experiments): *LUC7L2*, *KIF11*, and *ESR1* promoters. (***C***) ChIP analysis of well-known NRF1-regulated promoters (*FMR1*, *TFAM*, and *FXR2*) and negative controls (*GAPD* and *UMODL1* promoters) in MCF7 and U2OS cells. NRF1 (left panel), LSD1 (middle panel), and RNA PolII ChIP (right panel) analyses are shown. (***D***) LSD1 (orange or green), NRF1 (red), and H3K4me3 (black) ChIP-seq tracks in MCF7 cells (top) and mESCs (bottom) for well-known NRF1-regulated promoters: *TFAM* (left panel), *FMR1* (middle panel), and *FXR2* (right panel). LSD1 ChIP-seq data in mESCs was obtained from Whyte et al., 2012. Refseq annotation is shown at the bottom of each panel.
(PDF)

Figure S6 Binding of LSD1 to promoters of nuclear-encoded components of the mitochondrial electron transport chain. Top, left: scheme of multiprotein complexes involved in the electron transport chain (ETC) in the inner mitochondrial membrane. Panels: ChIP-seq tracks depicting loci of representative examples of nuclear-encoded components of the mitochondrial electron transport chain. LSD1 (orange or green), NRF1 (red), and H3K4me3 (black) ChIP-seq tracks from MCF7 cells (three top tracks) and mESCs (bottom track) are shown. LSD1 ChIP-seq data in mESCs was obtained from Whyte et al., 2012. Refseq annotation is shown at the bottom of each panel.
(PDF)

Figure S7 Fine co-localization of LSD1 and NRF1 in multiple mammalian promoters. ChIP-seq tracks of representative loci showing co-alignment of NRF1 (red) and LSD1 (orange) peaks. These particular examples were selected from the small list of promoters in which NFYB (blue) binds nearby NRF1 to help emphasize the good co-alignment between LSD1 and NRF1 using NFYB as reference. The H3K4me3 ChIP-seq track (black) is also shown as reference. Refseq annotation is shown at the bottom of each panel.
(PDF)

Figure S8 SiRNA-based screen in HEK293T cells to test KDM siRNA-mediated effects on luciferase expression under control of NRF1 and/or NFY motifs in the context of the sequence of their natural promoters. (***A***) Three sets of pGL2(basic) constructs were engineered to contain NRF1 (left), NFY (right), or NRF1 and NFY (middle) sites in the context of the sequence of their natural promoter cloned upstream the luciferase gene. The specific promoters were selected based on NRF1 and NFYB ChIP-seq data in MCF7 cells (promoter/gene names are listed in the figure), and all contain recognizable NRF1, NFY, or NRF1 and NFY motifs. (***B, C***) Hierarchical clustering of luciferase levels relative to control (scrambled) siRNA induced after the different siRNA treatments (listed in the figure). Constructs are listed on top: those containing NRF1 motifs are indicated by a red dot; while those containing NFY motifs are indicated by a blue dot. In *(B)*, it is shown the clustering analysis of the three NRF1- and the three NFY-regulated promoters. In *(C)*, it is shown the clustering analysis of the six promoters shown in (*B*) plus the three NRF1/NFY-regulated promoters.
(PDF)

Figure S9 Distribution of cardinal motifs and analysis of motif co-occurrences in promoters classified based on the mode of transcription initiation. *(* ***A*** *)* Comparison of co-occurrence matrixes of the seven cardinal motifs identified in this study (based on n = 21,000 promoters and −150/+50 bp regions; left panel) and the most-enriched motifs identified in a previous analysis by Fitzgerald et al., 2004 (based on n = 13,010 and −1,000/+500 bp regions; right panel). The values of motif co-occurrence in the right panel were directly derived from Fitzgerald et al., 2004. Co-occurrence log_2_ *p*-values are shown as a gradient of blue-to-red for positive-to-negative co-occurrences, and as white for no significant co-occurrence. *(* ***B*** *)* Analysis of cardinal motif preferences for promoters classified based on their mode of transcription initiation: focused (left) and dispersed (right). Number of promoters are indicated. We used 5′ RNA-seq data in MCF7 cells obtained from the DBTSS database (see Methods). The x-axis refers to log *p*-value of enrichment, and the y-axis includes motif names. *(* ***C*** *)* Co-occurrence matrixes of cardinal motifs in focused and dispersed promoters identified in MCF7 cells. Co-occurrence log_2_ *p*-values are shown as a gradient of blue-to-red for positive-to-negative co-occurrence, and as white for no significant co-occurrence.
(PDF)

Figure S10 Functional categories (GO terms) associated with genes differentially expressed in *LSD1* and/or *NRF1* depleted cells. *(* ***A–F*** *)* GO analysis of six of the eight classes of genes identified by expression microarray (as shown in **Figure 4D**–**4F**) that were affected by *NRF1* and/or *LSD1* siRNA treatments in U2OS cells: genes down-regulated (***A***) or up-regulated (***B***) by both treatments (*Class I* and *Class II*, respectively); genes exclusively down-regulated (***C***) or exclusively up-regulated (***D***) by *LSD1* siRNA (*Class V* and *Class VI*, respectively); and, genes exclusively down-regulated (***E***) or exclusively up-regulated (***F***) by *NRF1* siRNA (*Class VII* and *Class VIII*, respectively). The two remaining categories (*Class III* and *Class IV*) do contain a number of genes that is too low for reliable GO analysis (n = 18 and n = 35). *(* ***G*** *)* Analysis of NRF1 and LSD1 binding to active promoters regulating nuclear-encoded genes in MCF7 cells. LSD1 and/or NRF1-positive promoters are shown in the left cylinder, and LSD1 and NRF1-negative promoters are shown in the right cylinder. There are three subcategories in the first group: LSD1/NRF1 common-positive promoters (brown), LSD1 only-positive promoters (orange), and NRF1 only-positive promoters (red). Only H3K4m3-positive (active) promoters identified by ChIP-DSL in MCF7 cells and regulating genes in the category of ‘mitochondrion’ in the NCBI database were included in this analysis (those promoters of genes expressed in mitochondria, or H3K4me3-negative/inactive, or not present on the Hu20K array were excluded). Data based on H3K4me3, NRF1 and LSD1 ChIP-DSL experiments.
(PDF)

Figure S11 Protein interaction network of genes potentially regulated by LSD1. Cytoscape analysis of gene products associated with LSD1 binding in MCF7 cells (based on LSD1 ChIP-seq data). Full network analysis is shown on top, and two of the five high-score subnetworks are shown at the bottom. The full network and these two subnetworks show a clustering coefficient that is highly significant (*p*-value<0.001) compared with randomly generated networks with the same number of nodes. The three missing subnetworks in the figure included proteins that are included within the subnetwork shown in the right bottom of the figure. Functional categories are indicated next to each (sub)network. Gene names are indicated inside each node. Node color, node shape, and node size codes are indicated in the top right side of the figure.
(PDF)

Figure S12 Summary of proposed NRF1-LSD1 functional partnerships acting via −150/+50 bp regions. This summary only considers partnerships in which either NRF1 or LSD1, or both (based on microarray analysis upon *NRF1* and/or *LSD1* siRNA treatments) are functionally active. It excludes, therefore, the situation in which both NRF1 and LSD1 remain apparently inactive (based on the observation of no-effects by microarray upon *NRF1* and *LSD1* siRNA treatments). Classification (*Class* number) based on **Figure 4E and 4F**. See text for more details.
(PDF)

Table S1 NRF1 ChIP-seq peaks identified in MCF7 cells. Positions are expressed in hg18 coordinates.
(XLSX)

Table S2 NFYB ChIP-seq peaks identified in MCF7 cells. Positions are expressed in hg18 coordinates.
(XLSX)

Table S3 LSD1 ChIP-seq peaks identified in MCF7 cells. Positions are expressed in hg18 coordinates.
(XLSX)

Table S4 NRF1, NFYB, LSD1, and Sp1 ChIP-DSL positive promoters identified in MCF7 cells. NRF1, NFYB, and LSD1 ChIP-DSL experiments were performed in the Hu20K (in triplicate). The Sp1 ChIP-DSL experiment was performed in the Hu2K array (also in triplicate). We classified promoters as “4” when a positive promoter reached *p*<0.0001 (red cells), as “3” when reached *p*<0.001 (orange cells), as “2” when reached *p*<0.01 (yellow cells), or as “1” when *p*>0.01 (no binding, green cells). All results result from co-hybridization of ChIP and input samples. Note: the Hu20K array contains n = 20,000 promoters, but we limited our analyses to n = 17,288 promoters after eliminating n = 2,713 promoters (grey cells in the Excel file) in which the ChIP-DSL hybridization signal was too low or unreliable in at least one of the experiments.
(XLSX)
