# Supplementary figures and images for: Decoding a Signature-Based Model of Transcription Cofactor Recruitment Dictated by Cardinal Cis-Regulatory Elements in Proximal Promoter Regions
Source: PLoS Genet. 2013 Nov 7;9(11):e1003906. doi: 10.1371/journal.pgen.1003906 (PMC3820735; doi:10.1371/journal.pgen.1003906)

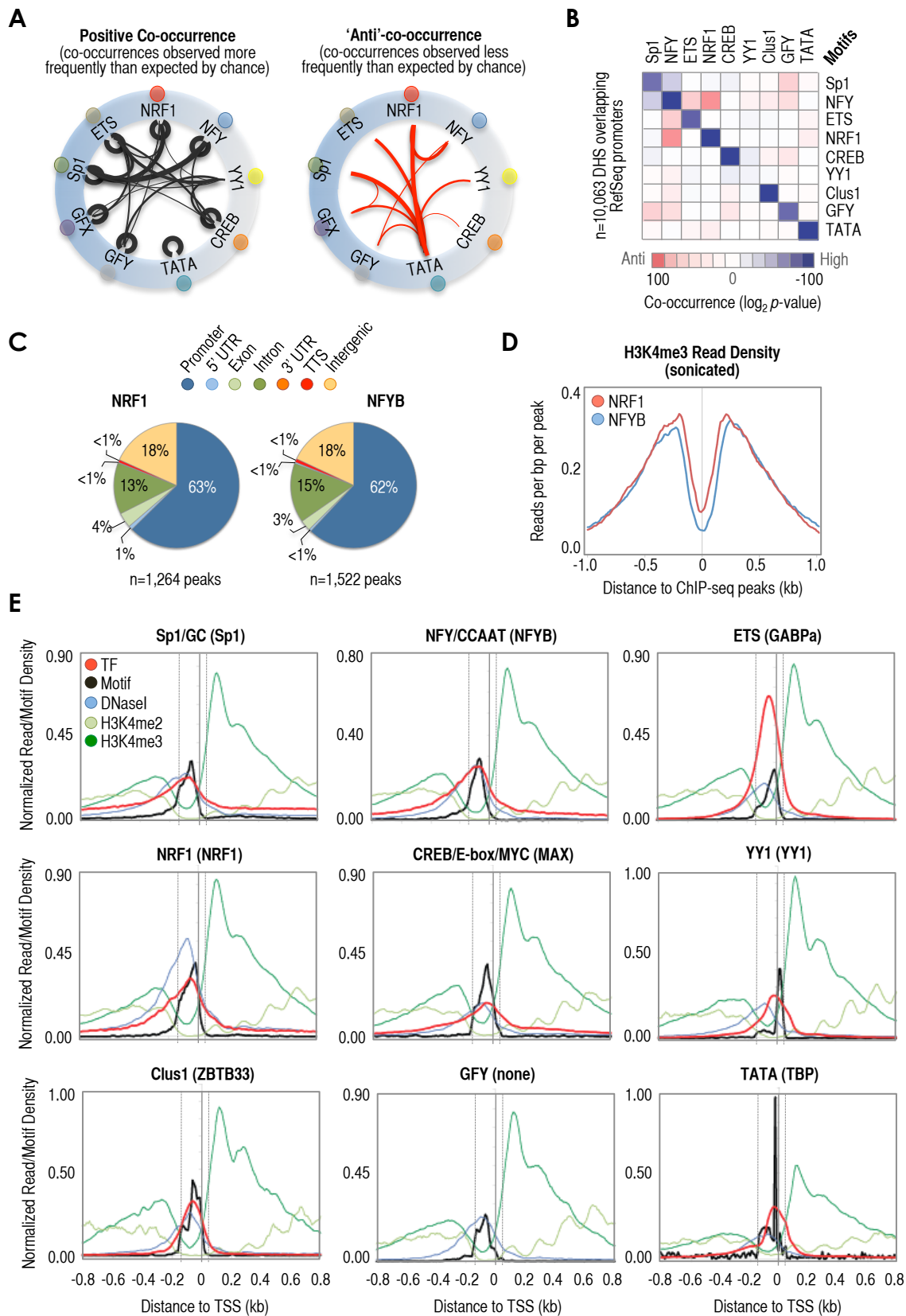

Benner et al. **Figure S1**

Supplement: Figure S1 — Cardinal motifs tend not to co-occur. (A) A summary (manually drawn with PowerPoint) of positive (left) and negative (right) motif co-occurrences identified in −150/+50 bp regions, based on Figure 1B . Connecting lines indicate a positive (black) or negative (red) co-occurrence between two cardinal motifs, and line thicknesses approximately correlate with levels of co-occurrence. (B) Co-occurrence matrix of cardinal motifs in n = 10,063 DHS regions defined experimentally by DNaseI-seq in human MCF7 cells that overlapped with RefSeq promoters. Co-occurrence log2 p-values are shown as a gradient of blue-to-red for positive-to-negative co-occurrence, and as white for no significant co-occurrence. (C) Distribution of NRF1 (left) and NFYB (right) ChIP-seq peaks with respect to the genome annotation. The numbers included in the pie charts refer to the fraction of peaks associated with each annotated region. The total number of peaks analyzed is also indicated. (D) Distribution of sequencing read density, based on H3K4me3 ChIP-seq, around NRF1 and NFYB ChIP-seq peaks (centre of the panel) in MCF7 cells. (E) Distribution of computationally predicted cardinal motif densities (black) and sequencing read density of: TF ChIP-seq data (red), DNaseI-seq data (light blue), H3K4me2 ChIP-seq data (light green), and H3K4me3 ChIP-seq data (dark green) with respect to TSS (vertical line at position 0). On top, the name of the specific motif and TF (in parenthesis) analyzed is shown. Doted vertical lines indicate −150 bp and +50 bp positions. DNaseI-seq, H3K4me2/3 ChIP-seq, NFYB ChIP-seq, GABPa ChIP-seq, NRF1 ChIP-seq, and MAX ChIP-seq data were obtained in MCF7 cells. Sp1, YY1, ZBTB33, and TBP ChIP-seq experiments were obtained in K562 cells. Source of sequencing datasets: all but H3K4me3, NRF1, and NFYB ChIP-seq experiments were produced by ENCODE and are publically available. (PDF) [file pgen.1003906.s001.pdf]

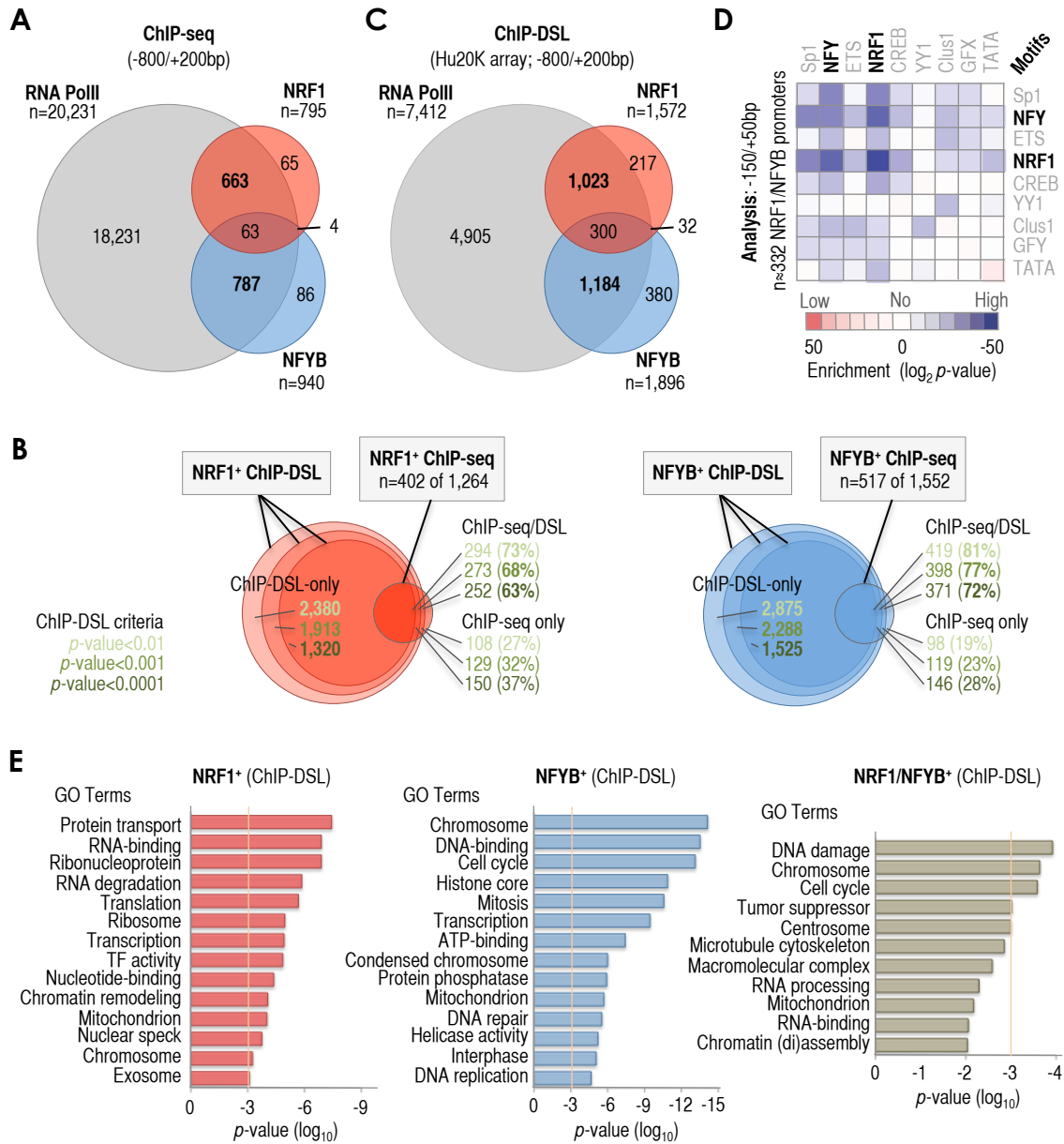

Benner et al. **Figure S2**

Supplement: Figure S2 — Poor rate of colocalization between cardinal TFs NRF1 and NFYB at −150/+50 bp regions and beyond. (A) Venn diagram depicting the overlap of NRF1 (red circle), NFYB (blue circle), and RNA PolII (grey circle) in MCF7 cells. NRF1 and NFYB data was based on ChIP-seq and peaks found between −800 bp and +200 bp relative to TSS. We considered as ‘overlap’ the coincidence of NRF1 and NFYB peaks in the same −800/+200 bp region. Also, we considered as ‘overlap’ the coincidence of RNA PolII peaks within ±1 kb of a TSS containing NRF1 or NFYB peaks at −8000/+200 bp. (B) Comparison of NRF1 (left) and NFYB (right) ChIP-seq peaks and ChIP-DSL positive hits in MCF7 cells. We compared the lists of −800/+200 bp genomic regions containing NRF1 or NFYB ChIP-seq peaks and the lists of NRF1 or NFYB ChIP-DSL positive hits (or promoters) to determine the number of coincident peaks/hits between both types of analyses. The analysis of ChIP-seq data was limited to the set of genomic regions present on the Hu20K array. We compared three levels of ChIP-DSL stringency based on p-values of positive hits: p<0.01, p<0.001, and p<0.0001. The percentage and number of promoters in each case are indicated. (C) Venn diagram depicting the overlap of NRF1 (red circle), NFYB (blue circle), and RNA PolII (grey circle) ChIP-DSL positive hits (p<0.0001) in MCF7 cells. The ChIP-DSL assay limits the analysis to −800/+200 bp regions relative to TSS. (D) Matrix of motif enrichment compared to background of the subset of n = 332 NRF1 and NFYB co-occupied promoters identified by ChIP-DSL. Motif analysis limited to −150/+50 bp regions. Motif enrichments higher than background are shown as a gradient of blue, while motif enrichments lower than background are shown as a gradient of red. No significant enrichment (equivalent to background) is shown as white. (E) Functional or GO analysis of genes with promoters occupied by NRF1 (left), NFYB (middle), or NRF1 and NFYB (right) based on ChIP-DSL analysis (p<0.0001). Selec [file pgen.1003906.s002.pdf]

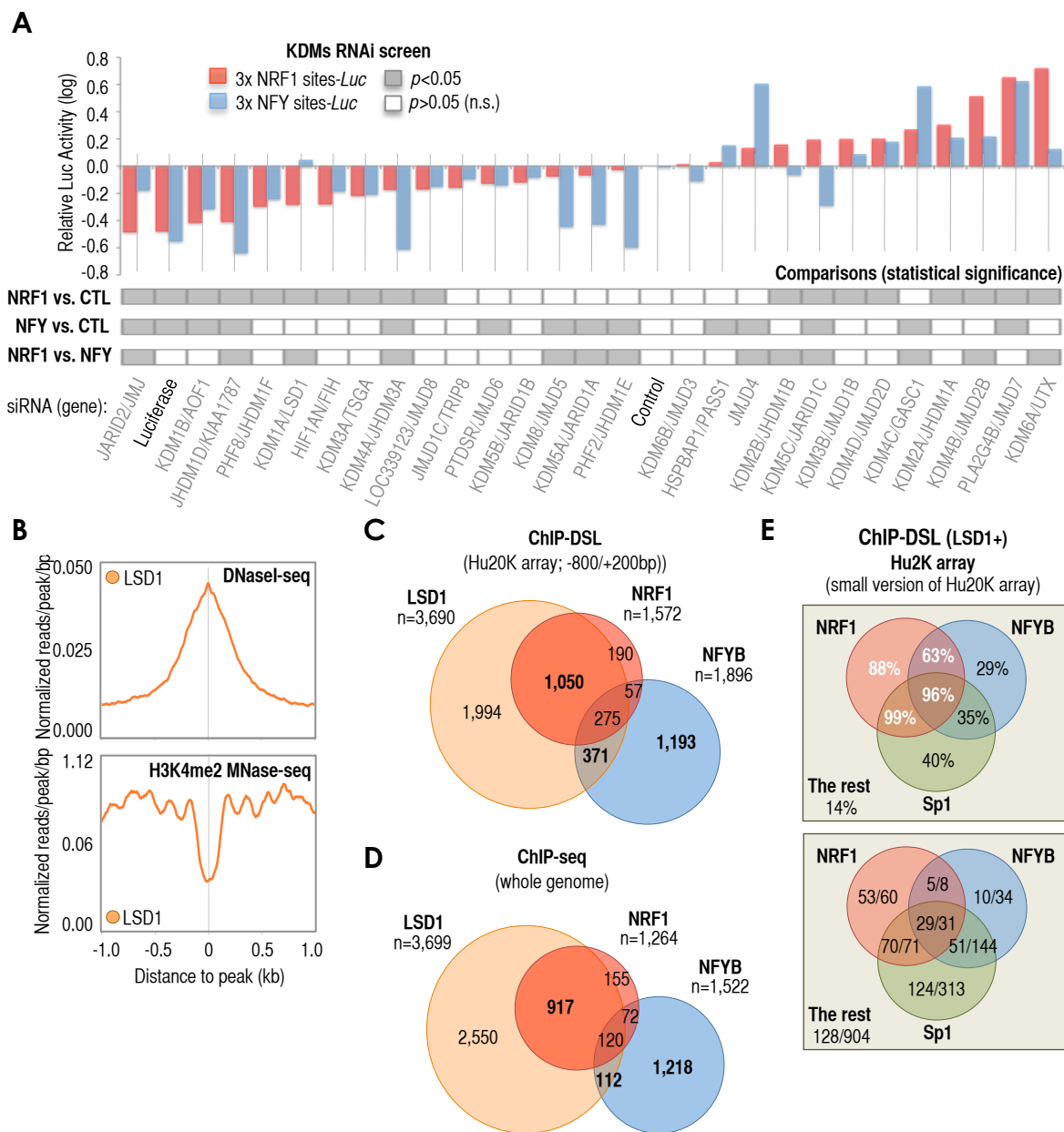

Supplement: Figure S3 — Strong co-association between cardinal TF NRF1 and cofactor LSD1 at genome-wide scale. (A) SiRNA screen based on the luciferase assay to identify KDMs (listed at the bottom) that may act selectively via 3×NRF1 sites (red) or 3×NFY sites (blue). A schematic overview of this screen and a summary of the results are shown in Figure 2A and Figure 2B , respectively. Selectivity for 3×NRF1 or 3×NFY sites is described in the figure legend of Figure 2B . Bottom: P-values≤0.05 are shown as grey boxes, p-value>0.05 are shown as white boxes. (B) Distribution of sequencing read density based on DNaseI-seq (top) and H3K4me2 MNase-seq (H3K4me2-marked nucleosomes, bottom) around LSD1 ChIP-seq peaks in MCF7 cells. (C) Venn diagram depicting the overlap of LSD1 (orange), NRF1 (red), and NFYB (blue) ChIP-DSL positive promoters in MCF7 cells based on the Hu20K array (−800/+200 bp). (D) Venn diagram depicting the overlap of LSD1 (orange), NRF1 (red), and NFYB (blue) ChIP-seq peaks in MCF7 cells without restriction of genomic localization (whole genome). (E) Percentage (top) and relative number (bottom) of LSD1-occupied promoters in NRF1- (red), NFYB- (blue), or Sp1- (green) occupied promoters in MCF7 cells, based on ChIP-DSL data. The percentage (top) and relative number (bottom) of LSD1-occupied promoters in cases that are not occupied by NRF1, NFYB, or Sp1 are also shown (large square). Analysis restricted to the n = 2,000 promoters present on the Hu2K array. The top panel shows percentage of LSD1-occupation relative to the total number of TF-positive promoters. The bottom panel shows absolute numbers of the same analysis. Data for Sp1 ChIP-DSL was obtained using the Hu2K array. Data for LSD1, NRF1, and NFYB ChIP-DSL was obtained using the Hu20K array, but their analyses were restricted to the set of promoters contained in the Hu2K array. Note: the Hu2K array is mostly constituted of cell cycle-regulated promoters, therefore, the percentages/frequencies of occupied promoters by the di [file pgen.1003906.s003.pdf]

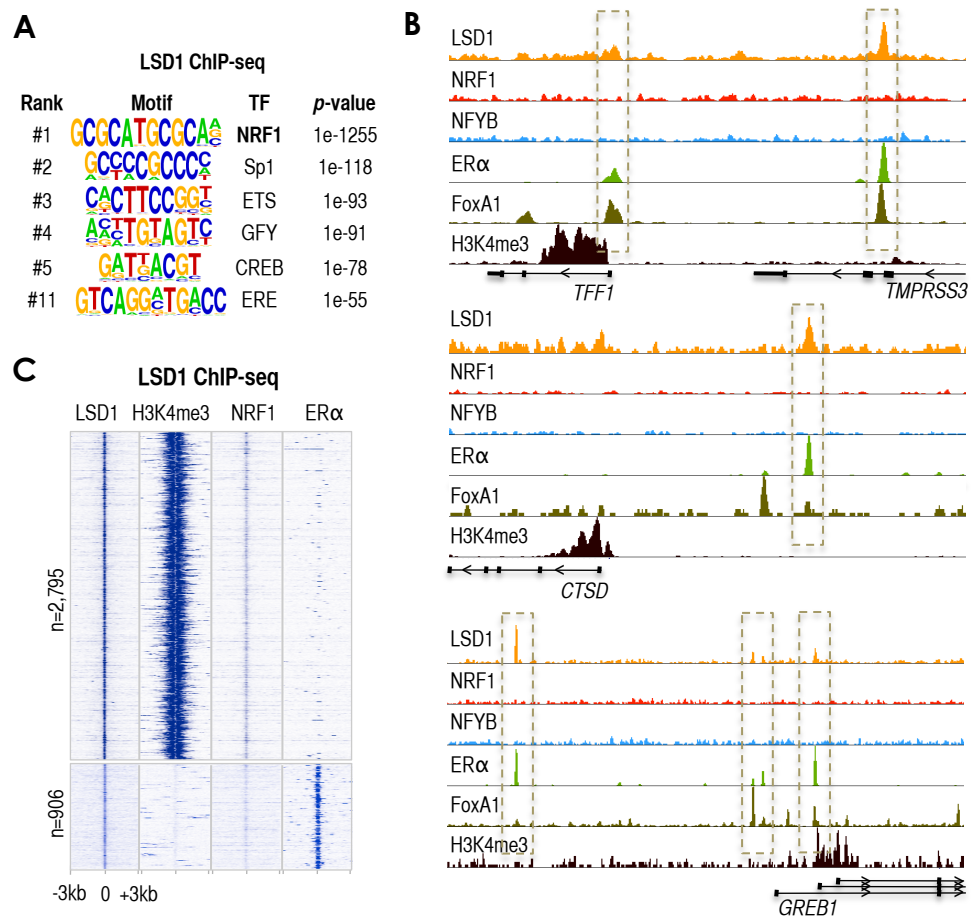

Supplement: Figure S4 — Strong co-association between LSD1 and NRF1 at proximal promoters and between LSD1 and ERα at distal sites in MCF7 cells. (A) Top-enriched motifs found in LSD1 ChIP-seq peaks in MCF7 cells identified by de novo motif discovery analysis. The panel includes: rank of motif enrichment, name of TFs associated with each motif, and p-value of enrichment. (B) LSD1 binding at genomic regulatory regions of representative examples of well-known ERα-regulated genes. The panel shows ChIP-seq tracks for LSD1 (orange), NRF1 (red), NFYB (blue), ERα (light green), FoxA1 (dark green), and H3K4me3 (black) at ERα-regulated loci: TFF1, CTSD, and GREB1. Refseq annotations are shown at the bottom of each panel. Coincident ERα and LSD1 peaks are enclosed in dotted boxes. (C) Heatmap analysis of LSD1, H3K4me3, NRF1, and ERα ChIP-seq signal ±3 kb around LSD1 ChIP-seq peaks (center of the columns). Two sets of LSD1 peaks were separately analyzed: LSD1 peaks nearby H3K4me3-marked regions (i.e. promoters; on top); and, LSD1 peaks nearby H3K4me3-negative regions (likely enhancers; at the bottom). (PDF) [file pgen.1003906.s004.pdf]

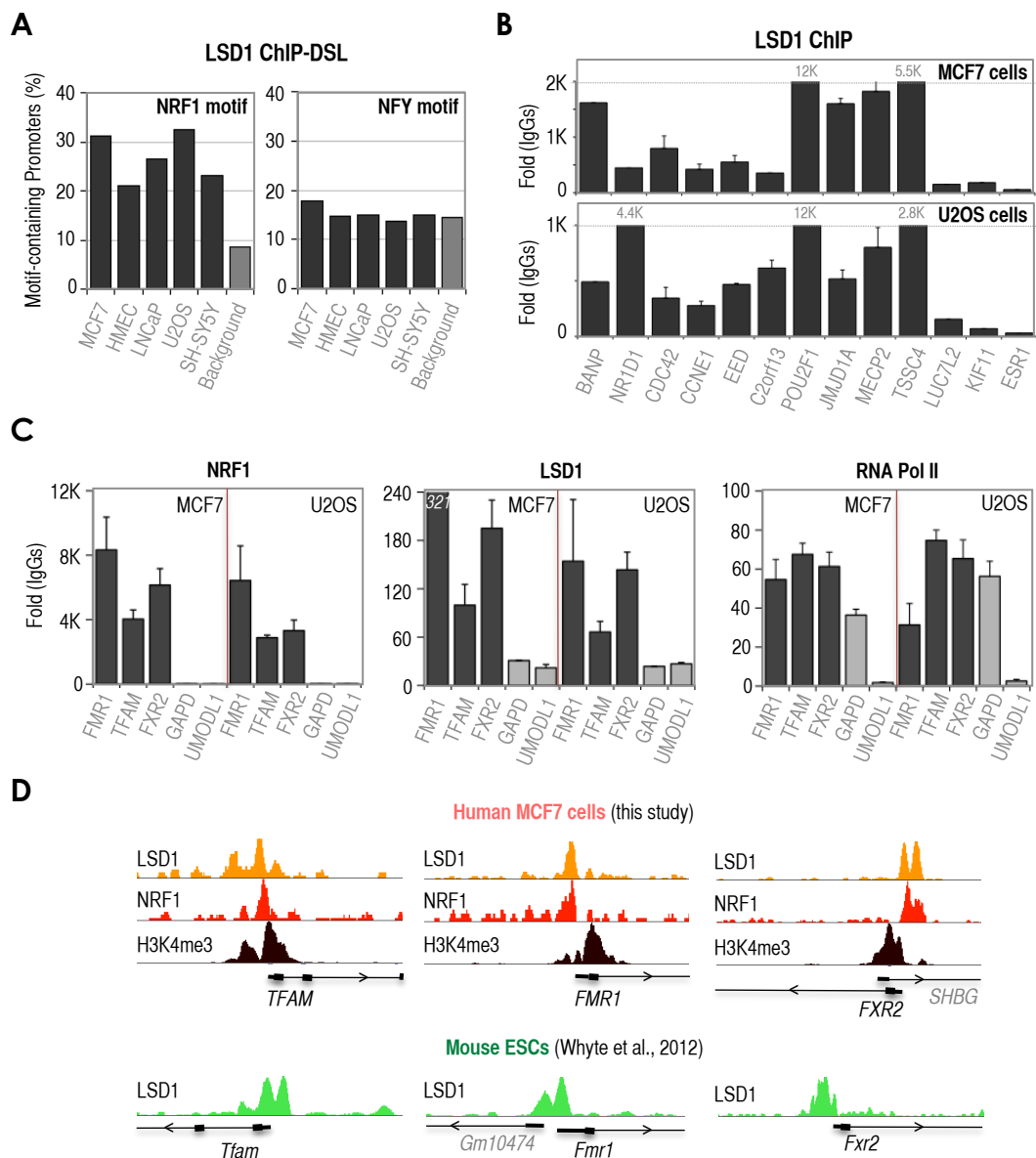

Supplement: Figure S5 — Strong binding co-association between NRF1 and LSD1 in different cell lines/types. (A) Percentage of NRF1 and NFYB occupied promoters that contain computationally predicted NRF1 or NFY motifs (left and right panels, respectively) in MCF7, HMEC, LNCaP, U2OS, and SH-SY5Y cells. Data based on ChIP-DSL analysis. ‘Background’ corresponds to the fraction of approximately 20,000 promoters in the Hu20K array that contain predicted NRF1 or NFY motifs. (B) ChIP validation of randomly selected LSD1 target promoters identified by ChIP-DSL in MCF7 cells (top), and ChIP analysis of the same promoters in U2OS cells (bottom). The results are shown as binding fold change over IgG signal. Three promoters were included as negative control (based on ChIP-DSL experiments): LUC7L2, KIF11, and ESR1 promoters. (C) ChIP analysis of well-known NRF1-regulated promoters (FMR1, TFAM, and FXR2) and negative controls (GAPD and UMODL1 promoters) in MCF7 and U2OS cells. NRF1 (left panel), LSD1 (middle panel), and RNA PolII ChIP (right panel) analyses are shown. (D) LSD1 (orange or green), NRF1 (red), and H3K4me3 (black) ChIP-seq tracks in MCF7 cells (top) and mESCs (bottom) for well-known NRF1-regulated promoters: TFAM (left panel), FMR1 (middle panel), and FXR2 (right panel). LSD1 ChIP-seq data in mESCs was obtained from Whyte et al., 2012. Refseq annotation is shown at the bottom of each panel. (PDF) [file pgen.1003906.s005.pdf]

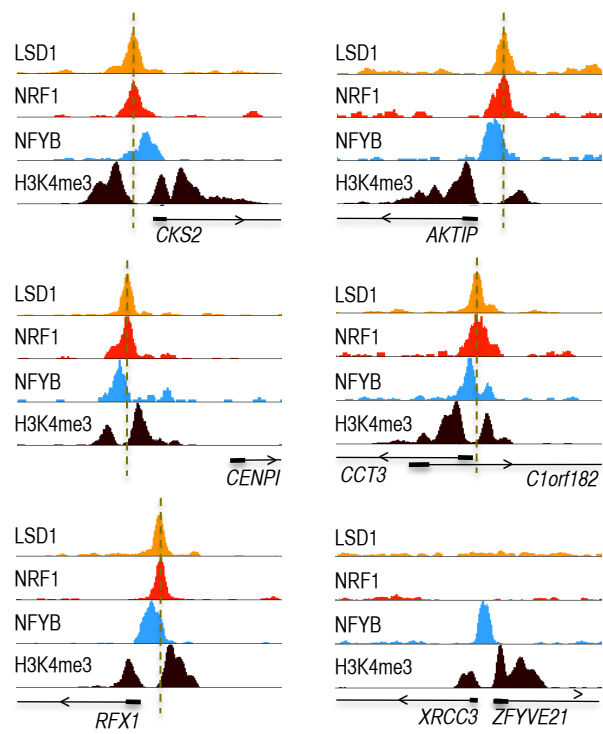

Supplement: Figure S7 — Fine co-localization of LSD1 and NRF1 in multiple mammalian promoters. ChIP-seq tracks of representative loci showing co-alignment of NRF1 (red) and LSD1 (orange) peaks. These particular examples were selected from the small list of promoters in which NFYB (blue) binds nearby NRF1 to help emphasize the good co-alignment between LSD1 and NRF1 using NFYB as reference. The H3K4me3 ChIP-seq track (black) is also shown as reference. Refseq annotation is shown at the bottom of each panel. (PDF) [file pgen.1003906.s007.pdf]

**A**

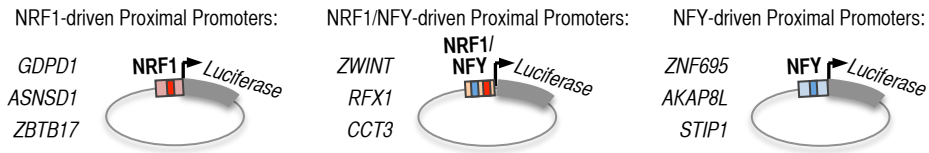

**B**

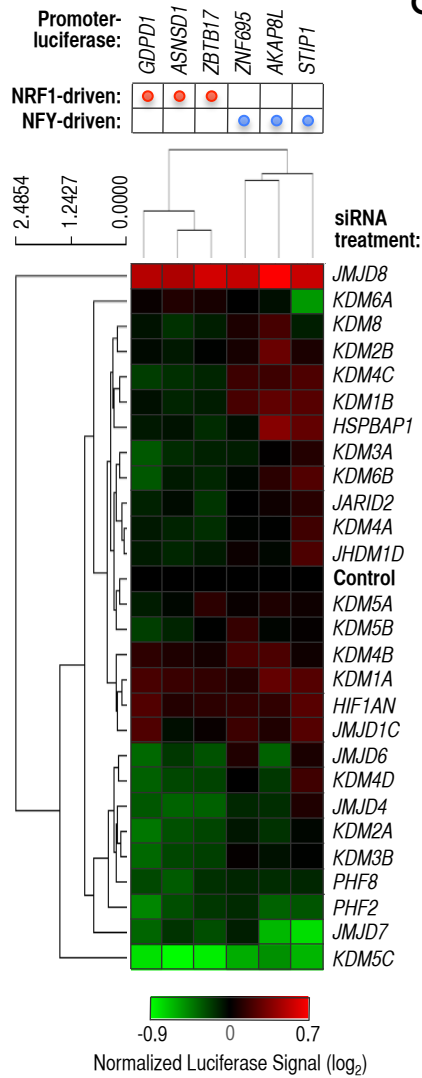

**C**

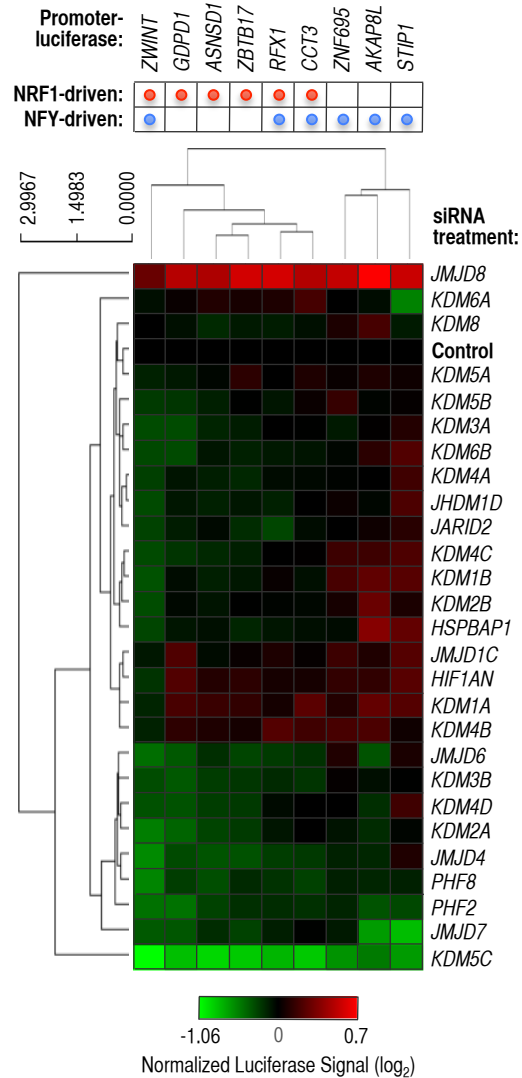

Supplement: Figure S8 — SiRNA-based screen in HEK293T cells to test KDM siRNA-mediated effects on luciferase expression under control of NRF1 and/or NFY motifs in the context of the sequence of their natural promoters. (A) Three sets of pGL2(basic) constructs were engineered to contain NRF1 (left), NFY (right), or NRF1 and NFY (middle) sites in the context of the sequence of their natural promoter cloned upstream the luciferase gene. The specific promoters were selected based on NRF1 and NFYB ChIP-seq data in MCF7 cells (promoter/gene names are listed in the figure), and all contain recognizable NRF1, NFY, or NRF1 and NFY motifs. (B, C) Hierarchical clustering of luciferase levels relative to control (scrambled) siRNA induced after the different siRNA treatments (listed in the figure). Constructs are listed on top: those containing NRF1 motifs are indicated by a red dot; while those containing NFY motifs are indicated by a blue dot. In (B), it is shown the clustering analysis of the three NRF1- and the three NFY-regulated promoters. In (C), it is shown the clustering analysis of the six promoters shown in (B) plus the three NRF1/NFY-regulated promoters. (PDF) [file pgen.1003906.s008.pdf]

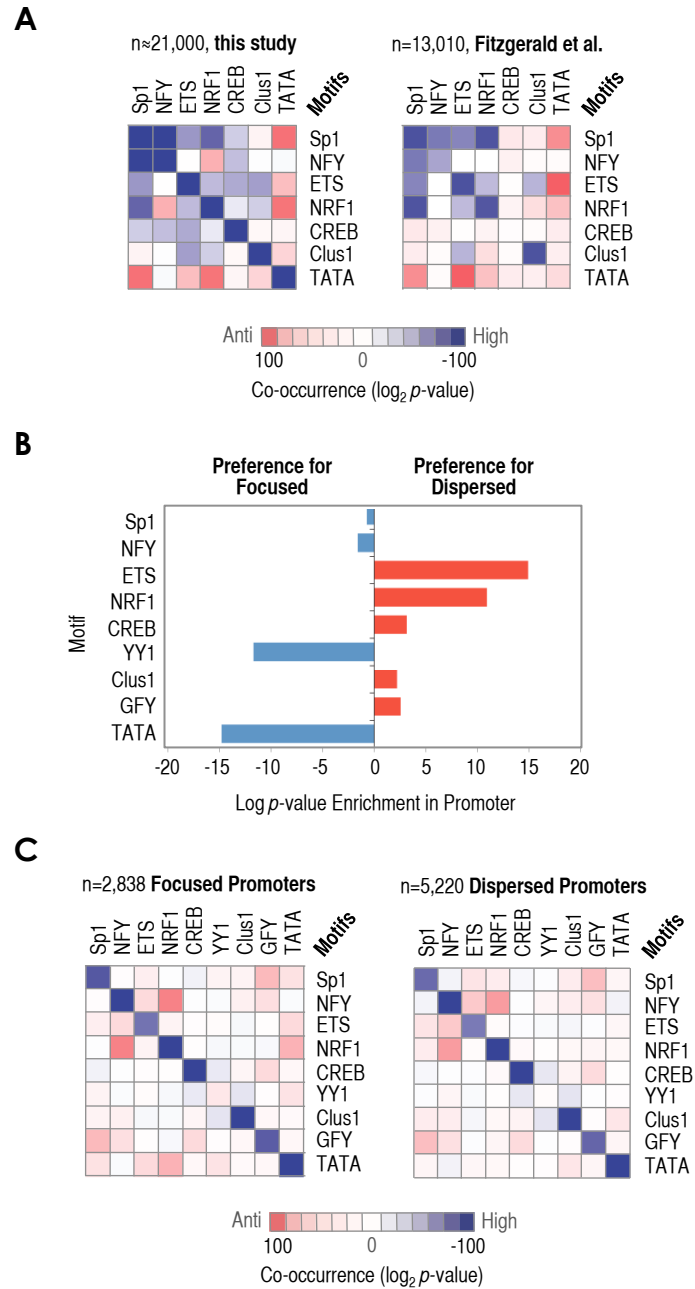

Supplement: Figure S9 — Distribution of cardinal motifs and analysis of motif co-occurrences in promoters classified based on the mode of transcription initiation. ( A ) Comparison of co-occurrence matrixes of the seven cardinal motifs identified in this study (based on n = 21,000 promoters and −150/+50 bp regions; left panel) and the most-enriched motifs identified in a previous analysis by Fitzgerald et al., 2004 (based on n = 13,010 and −1,000/+500 bp regions; right panel). The values of motif co-occurrence in the right panel were directly derived from Fitzgerald et al., 2004. Co-occurrence log2 p-values are shown as a gradient of blue-to-red for positive-to-negative co-occurrences, and as white for no significant co-occurrence. ( B ) Analysis of cardinal motif preferences for promoters classified based on their mode of transcription initiation: focused (left) and dispersed (right). Number of promoters are indicated. We used 5′ RNA-seq data in MCF7 cells obtained from the DBTSS database (see Methods). The x-axis refers to log p-value of enrichment, and the y-axis includes motif names. ( C ) Co-occurrence matrixes of cardinal motifs in focused and dispersed promoters identified in MCF7 cells. Co-occurrence log2 p-values are shown as a gradient of blue-to-red for positive-to-negative co-occurrence, and as white for no significant co-occurrence. (PDF) [file pgen.1003906.s009.pdf]

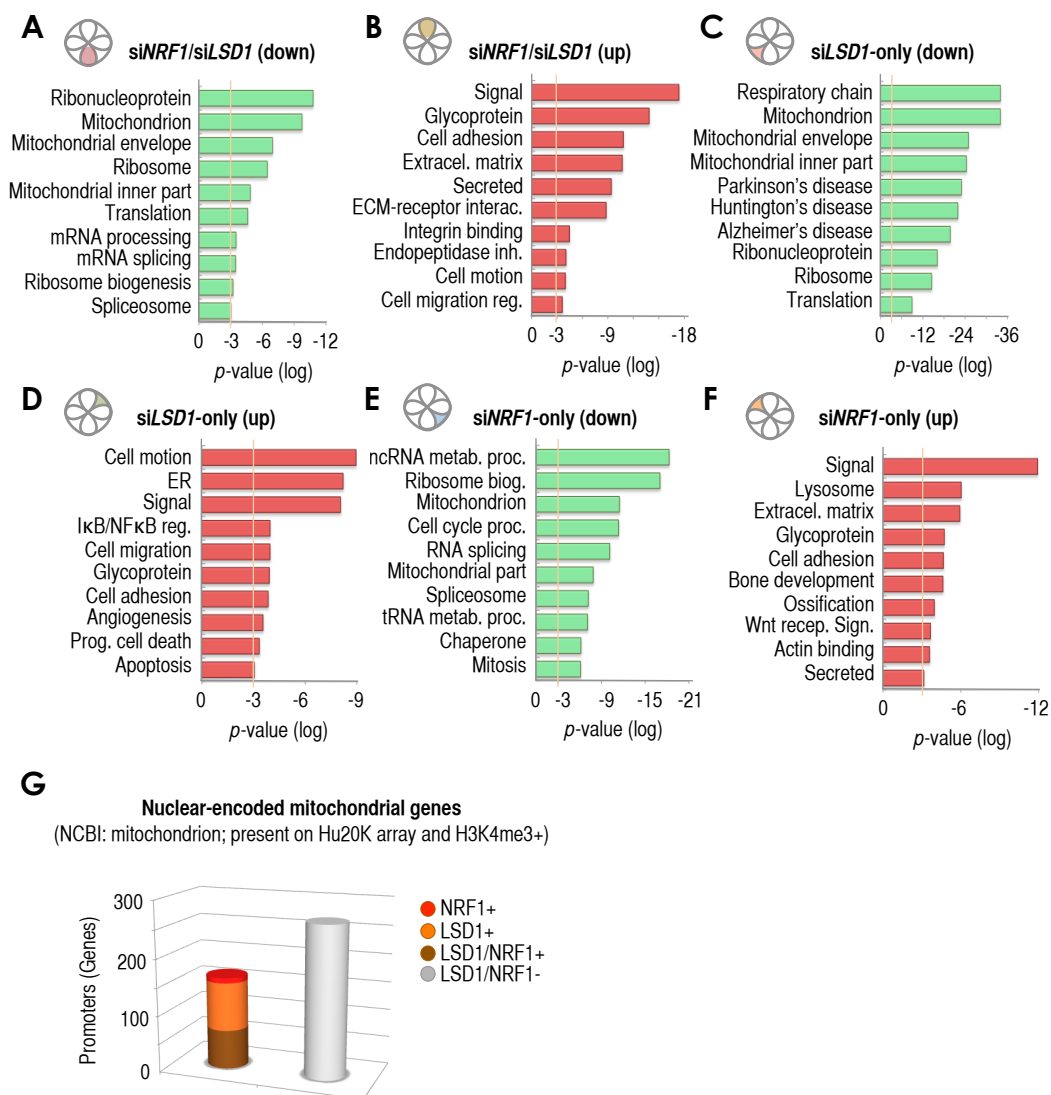

Supplement: Figure S10 — Functional categories (GO terms) associated with genes differentially expressed in LSD1 and/or NRF1 depleted cells. ( A–F ) GO analysis of six of the eight classes of genes identified by expression microarray (as shown in Figure 4D–4F ) that were affected by NRF1 and/or LSD1 siRNA treatments in U2OS cells: genes down-regulated (A) or up-regulated (B) by both treatments (Class I and Class II, respectively); genes exclusively down-regulated (C) or exclusively up-regulated (D) by LSD1 siRNA (Class V and Class VI, respectively); and, genes exclusively down-regulated (E) or exclusively up-regulated (F) by NRF1 siRNA (Class VII and Class VIII, respectively). The two remaining categories (Class III and Class IV) do contain a number of genes that is too low for reliable GO analysis (n = 18 and n = 35). ( G ) Analysis of NRF1 and LSD1 binding to active promoters regulating nuclear-encoded genes in MCF7 cells. LSD1 and/or NRF1-positive promoters are shown in the left cylinder, and LSD1 and NRF1-negative promoters are shown in the right cylinder. There are three subcategories in the first group: LSD1/NRF1 common-positive promoters (brown), LSD1 only-positive promoters (orange), and NRF1 only-positive promoters (red). Only H3K4m3-positive (active) promoters identified by ChIP-DSL in MCF7 cells and regulating genes in the category of ‘mitochondrion’ in the NCBI database were included in this analysis (those promoters of genes expressed in mitochondria, or H3K4me3-negative/inactive, or not present on the Hu20K array were excluded). Data based on H3K4me3, NRF1 and LSD1 ChIP-DSL experiments. (PDF) [file pgen.1003906.s010.pdf]

Summary of proposed NRF1-LSD1 functional partnerships acting via -150/+50bp regions

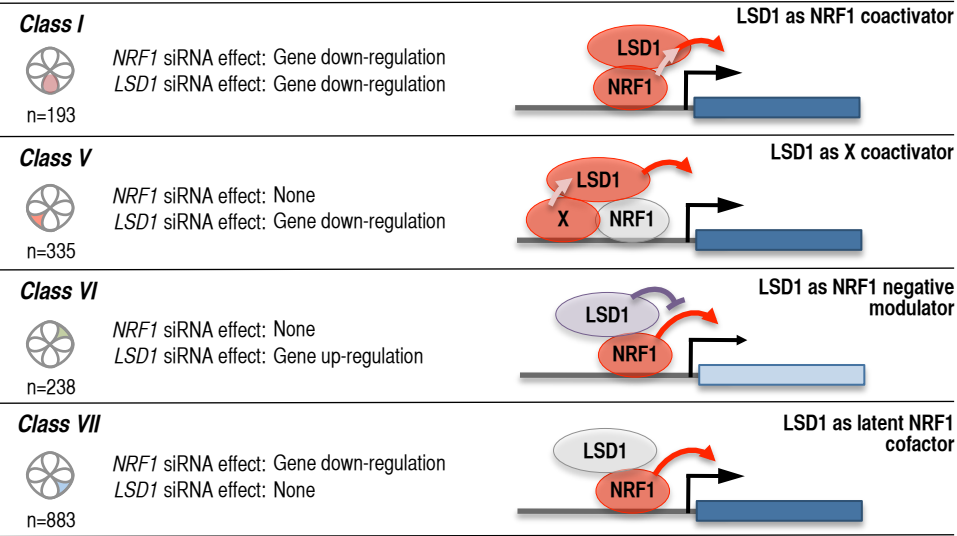

Supplement: Figure S12 — Summary of proposed NRF1-LSD1 functional partnerships acting via −150/+50 bp regions. This summary only considers partnerships in which either NRF1 or LSD1, or both (based on microarray analysis upon NRF1 and/or LSD1 siRNA treatments) are functionally active. It excludes, therefore, the situation in which both NRF1 and LSD1 remain apparently inactive (based on the observation of no-effects by microarray upon NRF1 and LSD1 siRNA treatments). Classification (Class number) based on Figure 4E and 4F . See text for more details. (PDF) [file pgen.1003906.s012.pdf]
