# Supplementary material for: Decoding a Signature-Based Model of Transcription Cofactor Recruitment Dictated by Cardinal Cis-Regulatory Elements in Proximal Promoter Regions
Source: PLoS Genet. 2013 Nov 7;9(11):e1003906. doi: 10.1371/journal.pgen.1003906 (PMC3820735; doi:10.1371/journal.pgen.1003906)
Supplement: Figure S6 — Binding of LSD1 to promoters of nuclear-encoded components of the mitochondrial electron transport chain. Top, left: scheme of multiprotein complexes involved in the electron transport chain (ETC) in the inner mitochondrial membrane. Panels: ChIP-seq tracks depicting loci of representative examples of nuclear-encoded components of the mitochondrial electron transport chain. LSD1 (orange or green), NRF1 (red), and H3K4me3 (black) ChIP-seq tracks from MCF7 cells (three top tracks) and mESCs (bottom track) are shown. LSD1 ChIP-seq data in mESCs was obtained from Whyte et al., 2012. Refseq annotation is shown at the bottom of each panel. (PDF) [file pgen.1003906.s006.pdf]

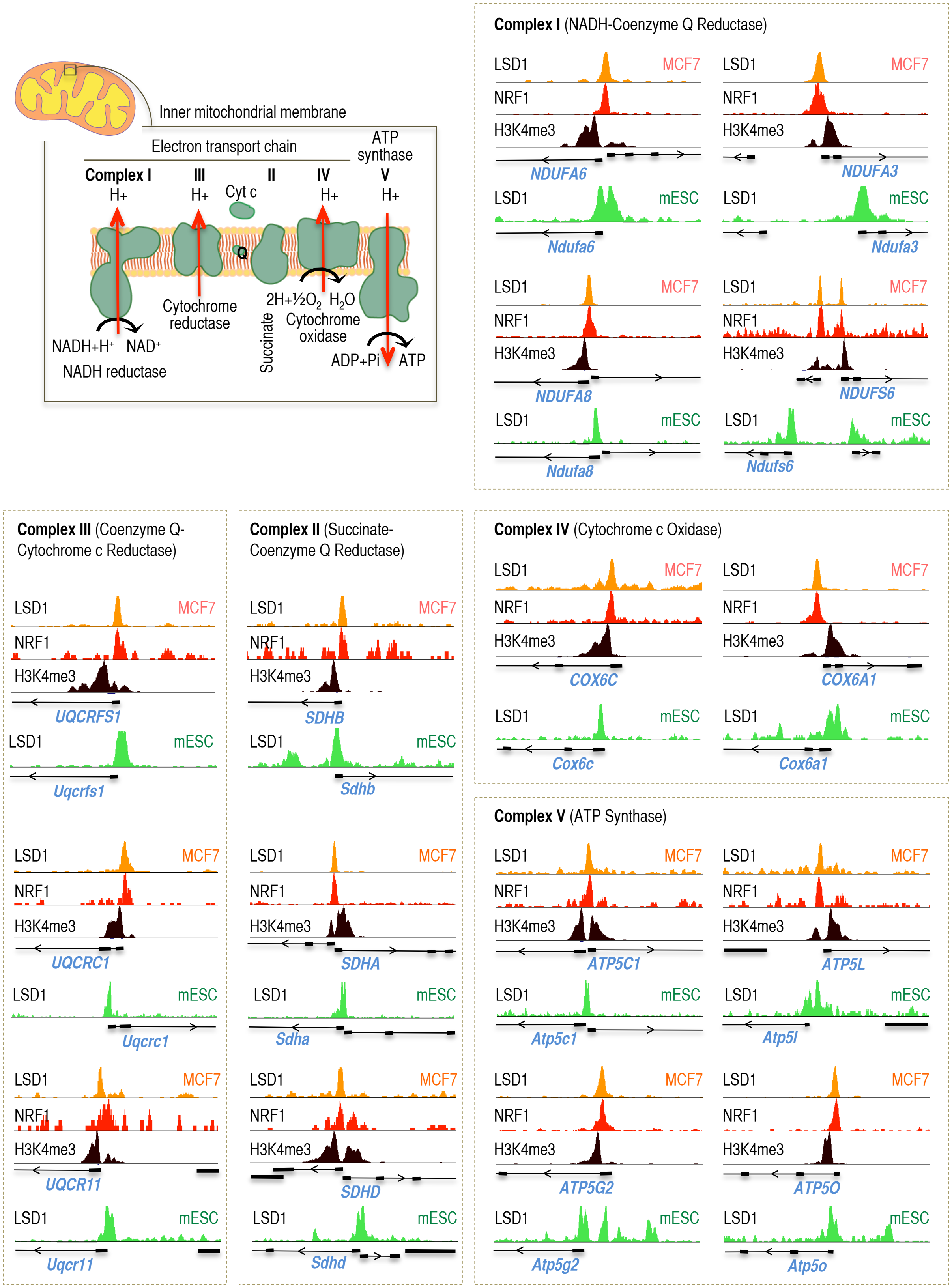

Benner et al. **Figure S6**
