# Supplementary material for: Decoding a Signature-Based Model of Transcription Cofactor Recruitment Dictated by Cardinal Cis-Regulatory Elements in Proximal Promoter Regions
Source: PLoS Genet. 2013 Nov 7;9(11):e1003906. doi: 10.1371/journal.pgen.1003906 (PMC3820735; doi:10.1371/journal.pgen.1003906)
Supplement: Figure S11 — Protein interaction network of genes potentially regulated by LSD1. Cytoscape analysis of gene products associated with LSD1 binding in MCF7 cells (based on LSD1 ChIP-seq data). Full network analysis is shown on top, and two of the five high-score subnetworks are shown at the bottom. The full network and these two subnetworks show a clustering coefficient that is highly significant (p-value<0.001) compared with randomly generated networks with the same number of nodes. The three missing subnetworks in the figure included proteins that are included within the subnetwork shown in the right bottom of the figure. Functional categories are indicated next to each (sub)network. Gene names are indicated inside each node. Node color, node shape, and node size codes are indicated in the top right side of the figure. (PDF) [file pgen.1003906.s011.pdf]

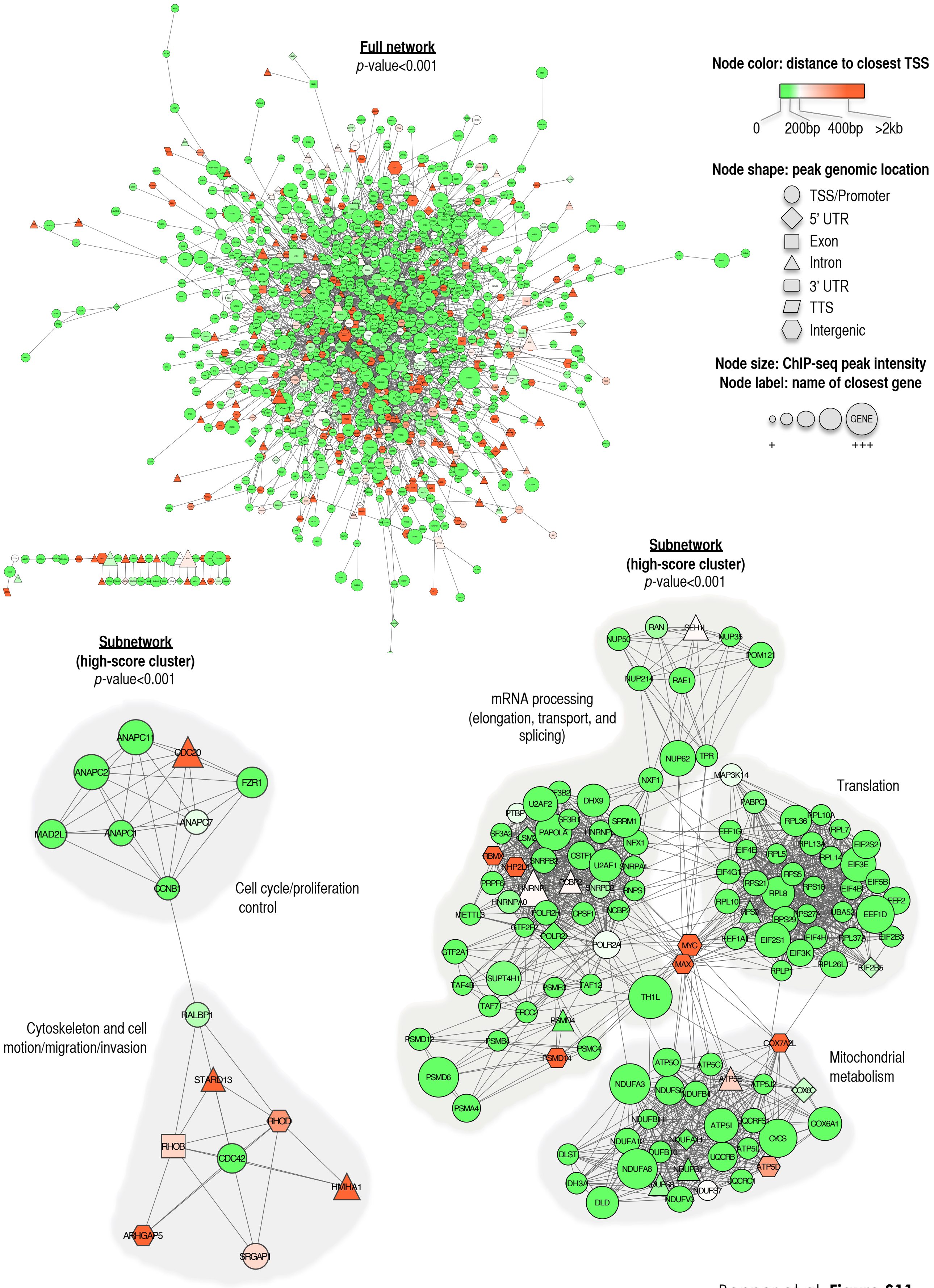

Benner et al. **Figure S11**
